# PREFER-IT: A transdisciplinary co-created framework to realise inclusive health AI

**DOI:** 10.1371/journal.pdig.0001096

**Published:** 2026-09-23

**Authors:** Patrícia Pita Ferreira, Sara Soriano Longarón, Wiam Bouisaghouane, Jetse Goris, Anne H. Hoekman, Balázs Markos, Benjamin Maus, Giorgia Pozzi, Hadi Hasan, Indre Kalinauskaité, Jonáh Stunt, Joosje D. Kist, Judith van der Elst, Katell Maguet, Liv Ziegfeld, Maarten Cuypers, Megan Milota, Michelle Habets, Sara Colombo, Špela Petrič, Steff Groefsema, Steven Warmelink, Elja Daae, Giovanni Briganti, Ildikó Vajda, Matias Valdenegro-Toro, Matthias Braun, Pieter Jeekel, Simone Goosen, Alex Schepel, Laxmie Ester, Riane Kuzee, Sophie de Klerk, Claudine Lamoth, Lisa Ballard, Mirjam Plantinga

**Affiliations:** 1 ELSA AI lab Northern Netherlands, Groningen, The Netherlands; 2 Department of Human Movement Sciences, University Medical Center Groningen, University of Groningen, Groningen, The Netherlands; 3 Department of Health Psychology, University Medical Center Groningen, University of Groningen, Groningen, The Netherlands; 4 Department of Neonatology, Beatrix Children’s Hospital, University Medical Center Groningen, University of Groningen, Groningen, The Netherlands; 5 Independent Researcher, Groningen, The Netherlands; 6 Department of Health Information Office, Information Management Healthcare, University Medical Center, University of Groningen, Groningen, The Netherlands; 7 STeP Research Group, Department of Transboundary Legal Studies, Faculty of Law, University of Groningen, Groningen, The Netherlands; 8 Department of Computer Science and Media Technology, Malmö University, Malmö, Sweden; 9 Faculty of Technology, Policy and Management, Delft University of Technology, Delft, The Netherlands; 10 Faculty of Law, University of Groningen, Groningen, The Netherlands; 11 Department of Global Public Health and Bioethics, Julius Center for Health Sciences and Primary Care, University Medical Center Utrecht, Utrecht, The Netherlands; 12 Department of Public Health and Primary Care, Leiden University Medical Center, Leiden, The Netherlands; 13 Department of Clinical Genetics, Erasmus Medical Centre, Rotterdam, The Netherlands; 14 Research Centre Art and Society, Hanze University of Applied Sciences, Groningen, The Netherlands; 15 Department for Human Machine Teaming, TNO, Soesterberg, The Netherlands; 16 Department of Primary and Community Care, Intellectual Disability and Health Group, Radboud University Medical Center, Nijmegen, The Netherlands; 17 Rathenau Instituut, Den Haag, The Netherlands; 18 Knowledge, Technology and Innovation Group, Wageningen University and Research, Wageningen, The Netherlands; 19 Department of Industrial Design Engineering, Delft University of Technology, Delft, The Netherlands; 20 Independent Artist, Amsterdam, The Netherlands; 21 Bernoulli Institute for Mathematics, Computer Science and Artificial Intelligence, Faculty of Science and Engineering, University of Groningen, Groningen, The Netherlands; 22 Research Group Digital Transformation, Hanze University of Applied Sciences, Groningen, The Netherlands; 23 AI Policy Lead, Ministry of the Interior and Kingdom Relations, Den Haag, The Netherlands; 24 Member of the ELSA Advisory Committee, The Hague, The Netherlands; 25 Unit of Computational Medicine and Neuropsychiatry, Faculty of Medicine, Pharmacy and Biomedical Sciences, University of Mons, Mons, Belgium; 26 Dutch Patient Federation, Utrecht, The Netherlands; 27 Department of Social Ethics, University of Bonn, Bonn, Germany; 28 Wellbeing and Healthcare Sector, AI Coalition for the Netherlands, Den Haag, The Netherlands; 29 Johannes Wier Foundation for Healthcare and Human Rights, Amsterdam, The Netherlands; 30 Ervaringsdeskundige/Expert by Experience, Sterk Uit Armoede, Haren, The Netherlands; 31 Ervaringsdeskundige/Expert by Experience, NALK (Netwerk Aanhoudende Lichamelijke Klachten), Deventer, The Netherlands; 32 National Institute for Health Research Biomedical Research Centre (Data, Health, and Society Theme), School of Primary Care, Population Sciences, and Medical Education, Faculty of Medicine, University of Southampton, Southampton, United Kingdom; 33 Department of Genetics and Data Science Center in Health, University Medical Center Groningen, University of Groningen, Groningen, The Netherlands; The University of Texas at Austin, UNITED STATES OF AMERICA

## Abstract

Artificial intelligence (AI) in healthcare holds transformative potential but risks exacerbating existing health disparities if inclusivity is not explicitly accounted for. This study addresses the disconnected discussions on inclusive health AI by developing a comprehensive framework, PREFER-IT. This framework is based on the outcomes of a five-day transdisciplinary co-creation workshop that involved 37 experts from diverse backgrounds, including healthcare, ethics, law, social sciences, AI, and patient advocacy, held in the Netherlands. For this workshop, we used design thinking and participatory methodologies to develop a framework for realising inclusive health AI. We identified three key challenges for realising inclusive health AI: integrating the lived experiences and stakeholder voices across the AI lifecycle, designing data collection practices that promote fairness and prevent inequalities, and fostering regulatory frameworks to uphold human rights and promote inclusivity. The analysis of participants’ perspectives informed the development of eight key thematic clusters of PREFER-IT: Participatory and co-design approaches (P), Representative and diverse data (R), Education and digital literacy (E), Fairness (F), Ethical and legal accountability (E), Real-world validation and feedback (R), Inclusive communication (I), and Technical interoperability (T). These elements were mapped across structural layers of AI (humans, data, process, system and governance) and the AI lifecycle to guide inclusive design, development, validation, implementation, monitoring, and governance. This framework fosters stakeholder engagement and systemic change, positioning inclusion as a guiding principle in practice. PREFER-IT offers a practical and conceptual contribution for how to include ethical, legal, and societal aspects when aiming to foster responsible and inclusive AI in healthcare.

## Author summary

Artificial intelligence (AI) is being used more and more in healthcare to improve diagnosis, treatment, and personalised care. However, if not designed carefully, these technologies can unintentionally increase existing inequalities and exclude certain groups from the benefits of these technologies. In our study, we brought together experts from healthcare, ethics, law, social sciences, and patient advocacy to explore how the application of AI in healthcare can be made more inclusive. During a five-day co-creation workshop in the Netherlands, we worked together to identify key issues and come up with practical solutions. We focused on three main areas: 1) Ensuring diverse voices are heard during the development of AI tools; 2) Making data collection fair and representative; and 3) Creating regulations that protect human rights. From the discussions of the workshop, we created the PREFER-IT framework, which outlines eight key principles for inclusive health AI: • **P**articipatory and co-design approaches, • **R**epresentative and diverse data, • **E**ducation and digital literacy, • **F**airness, • **E**thical and legal accountability, • **R**eal-world validation and feedback, • **I**nclusive communication, • **T**echnical interoperability. This framework helps guide developers, policymakers, and healthcare professionals in creating AI systems that are not only effective but also fair and respectful of all users. Our work emphasises the importance of involving patients and communities in shaping the future of AI.

## Introduction

The advances and applications of artificial intelligence (AI) in healthcare, referred to in this paper as *health AI*, have the potential to transform society. Health AI could improve diagnosis, personalise treatment, increase operational efficiency, optimise resources such as staff time, equipment, and hospital capacity, and improve patients’ experience [1–5]. Despite these possible benefits, health AI carries known risks: it may reinforce existing biases against historically disadvantaged populations, underserved groups, and individuals experiencing (multiple) layers of vulnerability [6], and it remains prone to errors and patient harm [7–9]. If left unaddressed, these risks could ultimately exacerbate health disparities [1]. Furthermore, health AI raises questions regarding accessibility, autonomy, equity, human rights, and inclusivity [1]. Inclusivity is a recognised principle in healthcare, although concerns remain that AI may reproduce or exacerbate disparities across patient and population groups [7,10]. Groups such as older adults, ethnic minorities, and individuals with disabilities may face disproportionate risks or limited benefits from AI innovations [1,10–12].

The literature shows a growing attention to issues of bias, trust, and fairness in AI [2,3,8,9,11,13–22], with different disciplines addressing these issues from complementary perspectives. Medical informatics research prioritises algorithmic fairness metrics [7,9] and technical bias mitigation [16,23,24], focusing on the iterative phases of the AI lifecycle, which encompass the design, development, validation, implementation, and monitoring of AI [2], while often not addressing social determinants of health and inequity. Ethics, legal, and social science literature emphasises autonomy, justice, and human rights, frequently lacking operationalisation into technical or clinical practice [25–27]. Clinical and public health research addresses data representativeness, patient outcomes, and health disparities, often concentrated in high-income contexts [7,10,26].

While each field contributes valuable insights to these issues, these contributions have largely evolved in parallel, resulting in a fragmented landscape of efforts that, while individually valuable, collectively limit the development of cohesive and actionable solutions. This fragmentation results in uneven attention to people experiencing (multiple) layers of vulnerability [6] (e.g., more emphasis on race and gender bias than on age, disability or their intersections [12,28]) and gaps across the AI lifecycle, with deployment, monitoring, and governance being less explored than, for example, data collection and model design [26]. Moreover, current approaches rarely engage with broader notions of inclusion, such as the social and structural contexts in which AI tools operate [12,29] or the systemic, participatory integration of marginalised voices and needs across the AI lifecycle.

To build on these diverse contributions, this paper adopts the lens of *inclusive health AI* to examine how principles of inclusion can be systematically embedded into the AI lifecycle and governance of AI in healthcare.

We define health inclusivity as the equal ability, opportunity, and right of all individuals, particularly those who are underserved, vulnerable, or otherwise disadvantaged based on identity and/or circumstances, to access health services, receive compassionate and high-quality care, and achieve equitable health outcomes, in ways that respect human dignity [13]. As a prerequisite for health inclusivity, AI systems must be designed and implemented to ensure equitable access and use across all population groups, regardless of attributes such as age, sex, gender, income, race, ethnicity, sexual orientation, disability, or other characteristics protected by human rights principles [1].

Some works, although not specifically focused on healthcare, have attempted to address this gap by exploring diversity and inclusion by design [9,24,25] or by advocating for stakeholder engagement in design and evaluation [13,30,31]. For example, Zowghi and da Rimini propose five pillars (humans, data, process, system, governance) for embedding diversity and inclusion by design across AI ecosystems. This approach extends beyond fairness metrics by urging the inclusion of diverse voices, contexts, and institutions [30]. Wang and Blok propose a multi-level framework that shifts from AI micro-level issues (e.g., dataset bias, algorithm transparency) to structural concerns, from meso (clinical and organisational) to macro (systemic and socio-political) levels, which shape AI’s broader impacts [25].

The FUTURE-AI guideline represents a first step, offering a structured, global consensus on trustworthy AI in healthcare across the AI lifecycle. While it addresses fairness, universality, and stakeholder engagement, its implementation of inclusivity remains limited: inclusivity is mainly viewed through bias mitigation and technical fairness metrics, rather than as a systematic design principle rooted in different contexts, lived experiences, and societal perspectives [2].

For healthcare specifically, the field remains fragmented, where valuable yet dispersed insights still need to be synthesised into a comprehensive, pragmatic framework for inclusive health AI. While technical and governance-oriented guidelines exist [2], and ethical principles have been articulated in policy documents [1,11], systematic approaches to realise inclusivity remain dispersed and underdeveloped. General frameworks on inclusivity are not always transferable to healthcare, given the high-stakes and sensitive nature of this domain [27,32]. Health AI tools have a direct impact on health outcomes, and the risks are substantial. Furthermore, healthcare is characterised by hierarchies and power dynamics, knowledge asymmetries between experts and patients, and the influence of professional and institutional norms [33–35]. Without explicit mechanisms for health inclusivity, these conditions can lead to the exclusion of voices and lived experiences.

This paper bridges this gap by presenting a set of co-created foundational requirements, named **PREFER-IT**, for the design, development, validation, implementation, monitoring, and governance of inclusive health AI. Developed through a transdisciplinary co-creation workshop held in the Netherlands, involving mostly European researchers and reflecting multiple disciplines and stakeholder perspectives, including patients, PREFER-IT provides a practical framework to realise health AI inclusivity. In this paper, we describe how PREFER-IT was developed.

## Methods

### Ethics statement

This study is based on qualitative anonymous data collected during the workshop *“Inclusive Medical AI: Exploring Diverse Perspectives”*. The workshop involved participatory design sessions with researchers and patient experts.

Ethical review and approval were not required for this study in accordance with the local legislation and institutional requirements. All participants provided informed consent to participate voluntarily and contributed to this publication to realise the dissemination of the collective insights. The study followed the ethical principles of the Declaration of Helsinki.

### Recruitment of participants

To explore the challenges of inclusive health AI and identify the requirements to address them, we organised a five-day transdisciplinary workshop, held from 10 to 14 February 2025 at the Lorentz Center in Leiden (The Netherlands). The event brought together 37 experts from different European countries, of whom six were organisers/facilitators, eight were (online) motivational speakers, and three were patient experts. The areas of expertise included the following categories: 1 – healthcare and public health; 2 – ethics, law, and policy; 3 – social sciences, psychology, humanities, and design; 4 – AI and data science; 5 – patient experts and advocacy. We employed a combination of purposive and snowball sampling to recruit participants, and experts were selected for their expertise in responsible AI and/or inclusive health(care). Attention was paid to ensuring diversity in disciplinary background, area of specialisation, seniority, geographical location, and gender, to foster multi-perspective dialogue (S1 Fig). The second day of the workshop included on-site patient experts from different underserved groups.

### Preparation and priority setting of the workshop

**Fig 1:**
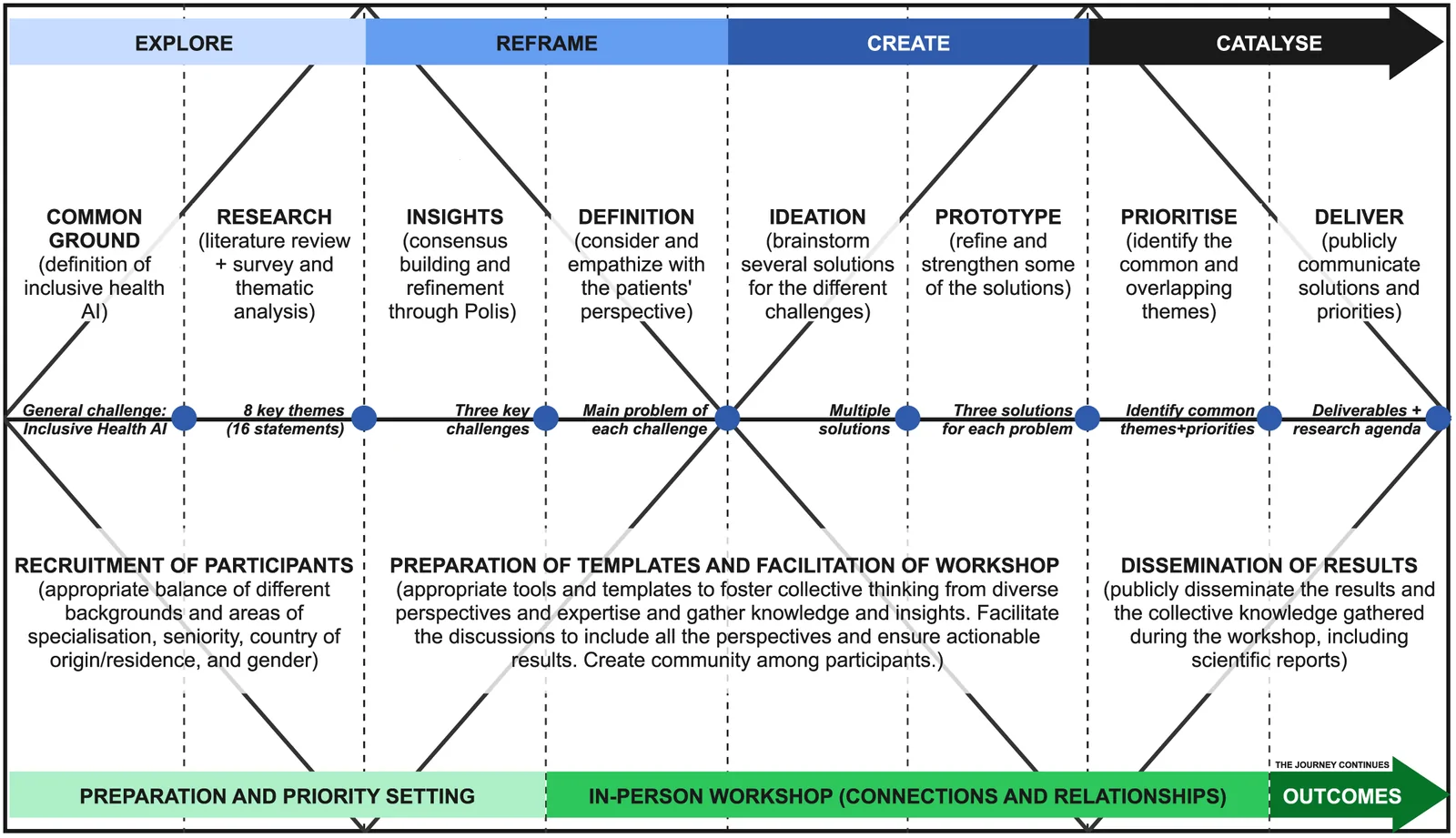
Structured workflow for the workshop. Adaptation of the double diamond model and the innovation and systemic design frameworks.

We used the design thinking methodology, adapting the double diamond model and the innovation and systemic design frameworks (Fig 1). Central to this method is the iterative phases for problem exploration and solution development, with the inclusion of all stakeholders [36–39]. The design thinking method was complemented by introducing a systemic approach for realising inclusive health AI, based on the different levels (micro, meso, macro), as proposed by Wang and Blok [25]. Additionally, the workshop aimed to integrate scientific insights with patient perspectives to promote transdisciplinary discussions, deepen understanding of the problem, and generate potential solutions or requirements (also known as ideation) for inclusive health AI. This structured approach facilitated knowledge exchange and collaboration across disciplines.

Fig 1 describes the framework for the different stages of the workshop, based on the design thinking methodology [36–38]. S2 Fig presents the adapted Wang and Blok framework, showing how the identified challenges of inclusive health AI and their proposed requirements relate across multiple levels [25].

In the preparation and priority-setting stages, participants completed a short anonymous online survey with three open-ended questions: how they understood inclusive health AI, what they considered necessary to realise it, and which topics should be prioritised in the workshop.

The first question informed our working definition of health inclusivity, which was presented in the introduction section (*Common Ground* stage) (During the workshop, we used the terms “medical AI” and “medical inclusivity”. Specifying “medical” rather than “health” did not limit the breadth of the discussion or the topics addressed, though realising the limitations of the word “medical” and following reviewer feedback, in alignment with PLOS Digital Health’s broader framing, we revised these to “health AI” and “health inclusivity” throughout the manuscript, to include public health, population health, community health, non-clinical contexts, and other settings where underserved populations most frequently encounter AI systems.). The second and third questions focused on the perceived needs and challenges associated with realising inclusive health AI. We conducted a thematic analysis of participants’ responses, which led to the identification of eight themes related to challenges for realising inclusive health AI (*Research* stage).

To further focus the discussion into a set of sub-challenges that could be effectively addressed during the in-person workshop, we performed a consensus-building exercise. For this, we started by drafting 16 statements (two per theme) from the thematic analysis, supported by ChatGPT-4o (OpenAI) for coding and phrasing. We then refined these statements through iterative cross-checking with the original survey data and the expertise of the research team. The resulting statements captured individual ideas related to the challenges of inclusive health AI and were subsequently used in Polis, an open-source digital platform for consensus building [40]. Polis collects and analyses opinions using machine learning, applying dimension reduction and clustering to identify average views and detect distinct opinion groups [40]. Participants could also contribute with their own statements, resulting in seven new anonymous submissions, all aligned with the original themes. A complete list of the statements and their corresponding themes is provided in Table 1.

**Table 1:** Themes and statements on inclusive health AI used in Polis.

Theme	Statement
***Bias in health AI***	1. Developing methods to minimise biases in AI systems.
	2. Understanding the effects of bias on AI outputs, such as unfair recommendations.
***Inclusivity in development (and implementation) processes***	3. Including diverse stakeholder voices throughout the AI lifecycle.
	4. Including the lived experiences of diverse groups in AI design and implementation.
	5. Address AI inclusivity in different stages of the AI lifecycle. ^a^
***Frameworks and standards***	6. Developing regulatory frameworks that address inclusivity in health AI.
	7. Creating regulatory frameworks interpretable for all stakeholders.
	8. Develop methods/frameworks to promote inclusivity for health experts working with AI to increase safe adoption (e.g., AI literacy). ^a^
	9. Make sure AI is at the service of patients. ^a^
	10. When developing and deploying healthcare AI, patients’ rights should be taken into account. ^a^
***Data representation and fairness***	11. Ensuring training datasets represent diverse groups.
	12. Assessing whether current data collection practices exacerbate inequalities.
***Impact assessment***	13. Investigating the societal impact of AI systems on diverse groups.
	14. Continuous evaluation of AI across diverse groups.
***Ethical, legal, and social considerations***	15. Identifying how health AI affects the human rights of diverse groups.
	16. Identifying how health AI affects public and personal values of diverse groups.
	17. As a matter of principle, I don’t think an AI tool should be used in the process of determining which topics are most pertinent. ^a^
***Future-proofing AI systems***	18. Incorporating AI advancements in the healthcare context.
	19. Monitoring and detecting problems and risks in AI systems before their release.
***Collaborative and holistic approaches***	20. Collaborating between different scientific disciplines.
	21. Using a systems approach.
	22. Address AI inclusivity at different ‘system’ levels (micro, meso, macro). ^a^
	23. Inventory and development of methods to include people in the process of AI development and implementation. ^a^

This table presents the eight key themes identified through thematic analysis of the survey responses, along with the initial 16 statements (two per theme) and the seven submitted anonymously by participants, drafted to inform the Polis deliberation.

^a^New statements added by the participants.

To include patient voices in the preparation and priority-setting stages, a focus group and a separate consensus-building exercise were conducted with a Patient and Public Involvement (PPI) group. During the consensus-building phase, participants used the Polis platform to share their perspectives and priorities in lay language. Their preferences were incorporated into the final set of sub-challenges on inclusive health AI prioritised for the workshop.

Participants of the workshop were asked to vote, through Polis, on each statement based on what they considered the most pressing challenges to address during the five-day in-person workshop (*Insights* stage).

Based on the consensus-building exercise through Polis (full results in S1 Table), we refined the sub-challenges considered a priority for the workshop. In addition to considering the level of agreement among participants and the feasibility of addressing the sub-challenges within the five-day format, we applied a set of criteria established after reviewing the Polis results. These criteria ensured that each selected sub-challenge was:

1. Distinctive, addressing unique and complementary aspects of the workshop’s overarching goal;
2. Balanced between human and technical dimensions, grounded in patient needs and lived experiences;
3. Interdisciplinary, suitable for engagement across diverse fields of expertise;
4. Aligned with the working definition of inclusive health AI, reflecting the core pillars of ability, opportunity, and dignity for equitable health outcomes;
5. Actionable and impactful, enabling actionable insights and identifying research gaps; and
6. Reflective of the PPI perspective, consistent with priorities identified by the PPI focus group.

Three challenges, each with a corresponding research question, were then identified to guide the work and discussions of the participants during the in-person workshop (*Insights* stage):

A. **Integrating lived experiences and stakeholder voices across the AI lifecycle:** *How can we ensure that the lived experiences of diverse groups and stakeholder voices are systematically included throughout the AI lifecycle?*
B. **Designing data collection practices to promote fairness and prevent inequalities:** *How can we ensure that data collection practices are representative, fair, and equitable and do not exacerbate inequalities while minimising biases in AI systems?*
C. **Fostering regulatory frameworks to uphold human rights and promote inclusivity:** *How can we ensure that regulatory and ethical frameworks are used in AI development and implementation to safeguard human rights, address ethical concerns, promote trust, and support inclusive practices across the AI lifecycle?*

### In-person workshop

The participants were divided into three groups during the in-person workshop, ensuring a balanced distribution of expertise. Each group was asked to collaborate in developing potential requirements and solutions for each of the three challenges related to inclusive health AI. The work of each group was guided by facilitators, using templates based on design thinking, which are summarised in S3 Fig.

Data were collected on sticky notes by the participants or by the facilitators on a working board for each group. Each day, the collected data from each group were uploaded as photographs to an online research drive and directly to a Miro board [41], ensuring that all participants could follow the group’s progress.

*During day one*, participants engaged in a structured, collaborative process to refine the three predefined challenges (*Definition* stage). Through facilitated brainstorming, each group deconstructed their assigned challenge into sub-problems, clustered them thematically, and mapped interconnections. To prioritise the most salient sub-problems, we used a consensus-based approach, complemented by dot-voting when needed (a participatory technique where participants visually cast votes with stickers to indicate their preferred options). Based on these, groups formulated preliminary problem statements, drawing on their disciplinary expertise and professional experience. Stakeholder mapping was conducted to identify key actors and relationships relevant to each challenge.

*On the second day,* three patient experts joined the workshop to co-create with participants. Each group collaborated with a patient to develop a patient persona and journey map, refining the initial problem statements from a patient-centred perspective. Personas (fictional yet evidence-informed representations of patient types) were used to capture diverse needs and experiences, while patient journey maps illustrated the steps and challenges individuals face within healthcare systems. This process enabled the group to explore how inclusive health AI could better support patients across different circumstances.

*The third day* was dedicated to the *Ideation* stage. Participants initially generated ideas, through silent ideation, for solving the challenges individually, then participated in group-based clustering, elaboration, and synthesis. They were encouraged to contribute ideas across groups, facilitating cross-pollination between challenges and enhancing transdisciplinary dialogue.

*On the fourth day,* participants focused on developing solutions (*Prototype* stage). Each group selected a subset of ideas generated during the ideation phase and elaborated on at least three in greater detail. This involved defining minimum implementation conditions, including roadmaps, technical and organisational requirements, enabling features, anticipated challenges, performance metrics, cost considerations, and stakeholder engagement strategies. The resulting prototypes were pitched to the whole group, allowing for iterative refinement based on collective feedback.

The personas and journey maps created on Day 2 served as a constant reference on each group’s work board. They were explicitly reviewed during Day 3 (*ideation*) and Day 4 (*prototyping*) through group discussions, dot-voting, and iterative refinements driven by inter-group presentations. Facilitators prompted groups to compare their solutions with the patient persona and journey map, ensuring that patient needs guided the evaluation criteria.

*The final day* focused on identifying common themes among the developed solutions and establishing shared priorities for advancing inclusive health AI through consensus (*Prioritise* stage). Participants collaboratively synthesised overlapping themes and reflected on missing elements. This concluding session consolidated workshop outcomes, with a strong emphasis on sustained patient involvement and the realisation of inclusivity principles throughout the AI lifecycle.

*After the workshop*, we analysed the raw data. The most important findings and the collective insights from the workshop are presented in the results section (*Deliver* stage).

### Declaration of generative AI and AI-assisted technologies

During the workshop preparation, the authors used ChatGPT-4o (OpenAI) to help conduct a preliminary thematic analysis of the survey sent to the participants and draft the preliminary initial statements fed into Polis. ChatGPT-4o (OpenAI) was also used to simplify and annotate the code to analyse raw data and generate S1 Table and S1 Fig in R statistical software (version 4.4.3; R Foundation for Statistical Computing). While preparing this manuscript, the authors used AI-assisted tools (e.g., Grammarly) to check grammar and spelling and improve readability. After using these tools, the authors reviewed and edited the content as needed and take full responsibility for the content of the published article.

## Results

**Fig 2:**
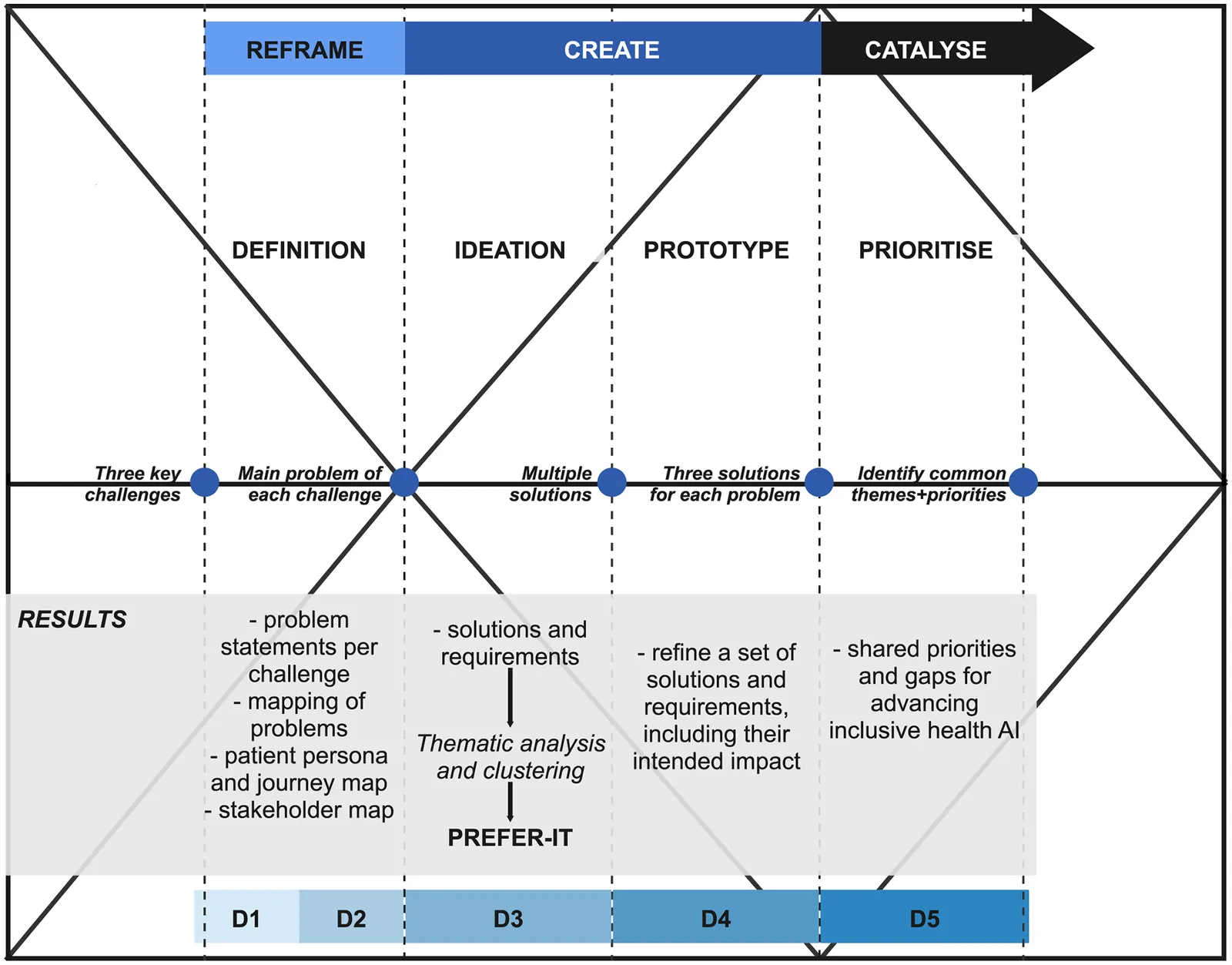
Results of the workshop summarised by day (D). The collective insights and main outcomes of the in-person workshop, by day.

The workshop and the development of the PREFER-IT framework followed an iterative co-creation process involving the stages of *definition*, *ideation*, *prototype*, and *prioritisation*. This process culminated in a final phase of the *PREFER-IT framework consolidation* based on the thematic analysis and clustering of the workshop data. The collective insights and results from the in-person workshop are summarised in Fig 2.

### Definition

Table 2 summarises the problem clusters, core problem framing and principal stakeholders for each challenge (the complete point-of-view problem statements and full stakeholder maps are provided in S2 Table). Across all three predefined challenges, participants identified a set of converging concerns regarding the inclusivity of AI in healthcare (*Definition* stage).

**Table 2:** Summary of the definition stage for each challenge.

	A – Integrating lived experiences and stakeholder voices across the AI lifecycle	B – Designing data collection practices to promote fairness and prevent inequalities	C – Fostering regulatory frameworks to uphold human rights and promote inclusivity
**Problem clusters**	1. Power; 2. Frameworks; 3. Representation	1. Need/incentives for data; 2. Society; 3. Parameter conditions; 4. Processing	1. Semantics; 2. Trust and transparency; 3. Multilevel/systems thinking; 4. Power structure; 5. System development; 6. Policy; 7. Human rights; 8. Morality
**Core problem framing**	Patients and underserved groups are insufficiently engaged across the AI lifecycle, while developers and researchers lack the interaction and methods needed to identify and act on their needs, values, and emotions.	No shared process exists for co-creating decisions about what data are collected; current practices underserve hidden and hard-to-reach populations, and developers lack the autonomy to prioritise purposeful collection	Economic efficiency pressures demand stronger oversight; developers and regulators face uncertainty in achieving compliance; and framework developers work from a limited view of the relevant levels and voices.
**Principal stakeholders**	Patients, developers, HCP, researchers/academia, ethicists, government, business and lobby organisations	Funding agencies, HCP, researchers, developers, policy makers, patients, community and citizens, technology providers	Policy makers and regulatory bodies, AI developers, HCP, hospital boards, patients and patient organisations, citizens, private companies and the pharmaceutical industry, researchers

Summary of the problem clustering, core problem framing, and principal stakeholders (in no particular order) identified for each challenge. Problem statements were originally formulated as design-thinking point-of-view statements of the form “We assume that for [actor], there is [need]… to realise inclusive health AI”; the complete statements and full stakeholder maps are provided in S2 Table. AI – artificial intelligence; HCP – healthcare professionals.

Challenge A (integrating lived experiences and stakeholder voices) was organised into three problem clusters: power, frameworks, and representation, from which participants derived three actor-specific problem statements. They posited that business and healthcare companies have insufficient interaction with patients to create personalised AI solutions; that underrepresented patients lack the participation and inclusion, within health AI, needed to secure their autonomy and accessibility; and that AI researchers face difficulty in identifying and categorising patients’ needs, knowledge, values, and emotions to realise health AI inclusivity.

For Challenge B (designing data collection practices), participants identified four clusters: need and incentives for data, society, parameter conditions, and processing. They argued that healthcare users are rarely offered a process, protocol, or prerequisite of co-creation through which to determine what data are collected; that hidden and difficult-to-reach populations, such as groups with low socioeconomic status (SES) or intellectual disabilities, are poorly served by existing data practices and including these data require alternative approaches to identify, collect, and process relevant data and to engage them meaningfully in research; and that AI developers lack the decisional autonomy to prioritise purposeful data collection to realise health AI inclusivity.

Challenge C (fostering regulatory frameworks) identified eight clusters: semantics, trust and transparency, multilevel and systems thinking, power structure, system development, policy, human rights, and morality. Participants argued that healthcare professionals (HCP), policymakers, and patients operate under economic efficiency pressures that require stronger oversight; that AI developers and regulatory bodies face uncertainty about how to comply with regulations and need practical, actionable guidance; and that those who develop regulatory frameworks work from a limited and biased view of the relevant levels and voices, and need to embrace more creative and exhaustive stakeholder analysis to realise inclusive health AI.

A central theme across challenges was the presence of structural and procedural barriers that disempower specific stakeholders (especially patients, disadvantaged and underserved groups, and individuals experiencing (multiple) layers of vulnerability, but also AI developers), leading to inequities in participation and decision-making. The three groups also identified the absence of integrated, practical, and inclusive frameworks as a significant limitation across all challenges: from a lack of methods to categorise patients’ values and needs (challenge A), to fragmented approaches to data co-creation and processing (challenge B), to uncertainty around regulatory compliance (challenge C). They also highlighted the need to improve the representation, engagement, and inclusion of diverse populations in AI design, development, validation, implementation, monitoring, and governance. Particular attention is required for underserved patients and people experiencing (multiple) layers of vulnerability to ensure that all voices are meaningfully considered.

Another common finding was the need for AI practices to be more responsive to lived experiences, contextual differences, and social determinants of health. During the stakeholder mapping exercise, participants identified the stakeholders most relevant to addressing each challenge, including policymakers, HCP, developers, patients, industry, and researchers. While the exact configuration varied across challenges, there was a common emphasis on involving diverse stakeholders from different sectors and fostering partnerships (Table 2).

### Ideation

**Fig 3:**
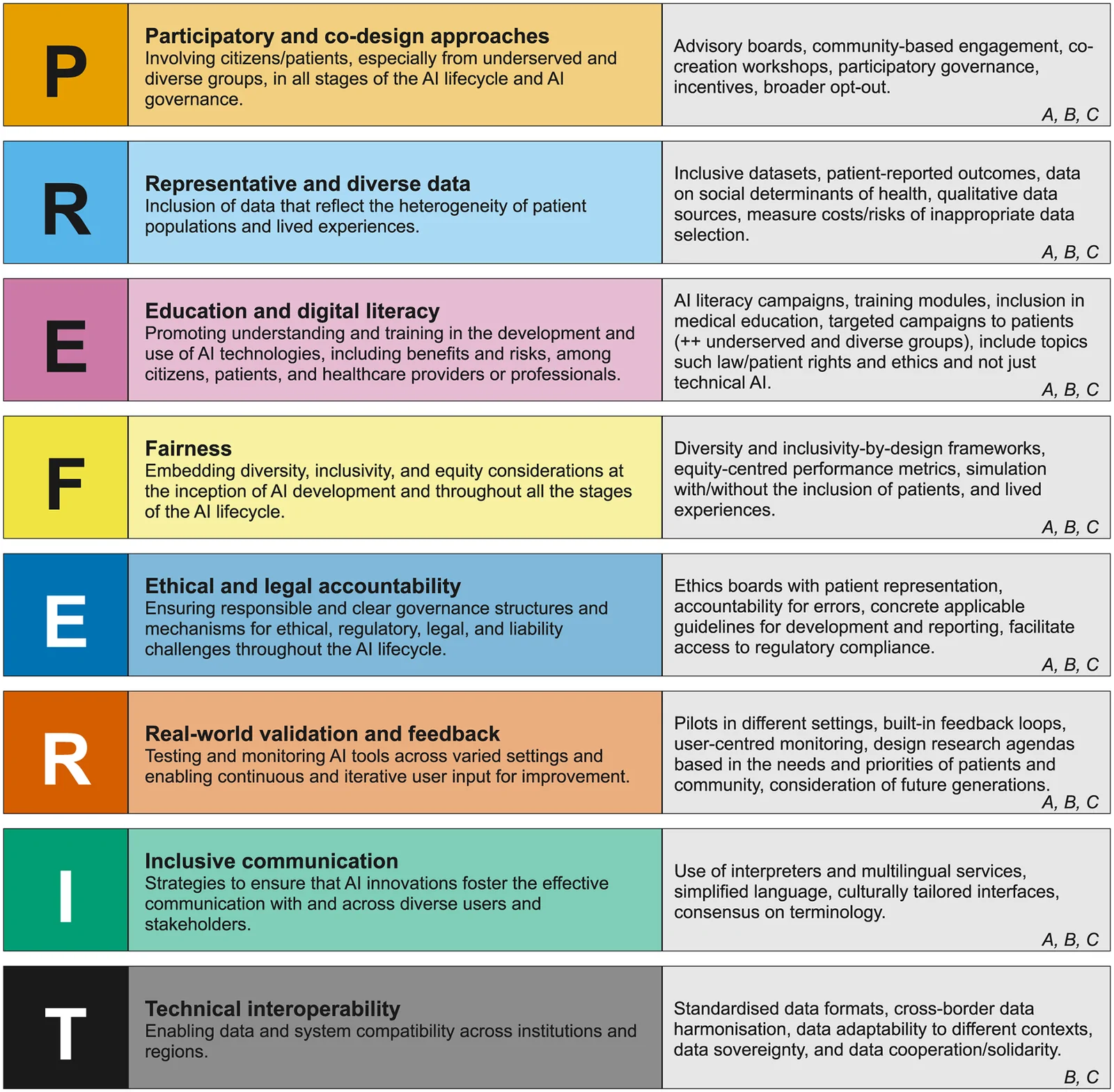
PREFER-IT framework. How PREFER-IT originated from the thematic clusters that emerged after ideation for solutions and requirements for inclusive health AI, and clustering across the three challenges. The colours of PREFER-IT follow the 8-colour Okabe-Ito palette [42,43] and are used for categorical identification only, with no implied ordering or priority. In grey, the figure presents concrete solutions for implementing the PREFER-IT requirements across the workshop challenges (A, B, C).

The *Ideation* stage led to the development of more than 180 solutions or requirements for inclusive health AI. Through a thematic analysis and clustering conducted after the workshop, we synthesised these into eight key thematic clusters, which informed the development of the **PREFER-IT** framework (Fig 3; a graphical overview of PREFER-IT is provided in S4 Fig).

- *P* stands for *Participatory and co-design approaches*. Participants consistently emphasised the importance of involving underserved and diverse communities, including patient representatives, throughout the entire AI lifecycle.
- *R* represents the need for *Representative and diverse data*. Participants stressed the importance of integrating data that accurately reflects the heterogeneity of patient populations and incorporates the lived experiences of patients.
- *E* refers to *Education and digital literacy* for enabling critical engagement with AI technologies. Participants highlighted the need for targeted educational initiatives to enhance the understanding of AI among patients, HCP, and the broader public, addressing both the potential benefits and risks.
- *F*, for *Fairness*, encapsulates the demand for the proactive integration of diversity, inclusivity, and equity considerations from the inception of AI development, and throughout all stages of the AI lifecycle.
- The second *E* denotes *Ethical and legal accountability*. Participants emphasised the need for clear governance structures and accountability mechanisms to address ethical, legal, and regulatory challenges throughout the AI lifecycle.
- *R* also stands for *Real-world validation and feedback*. There was a broad consensus on the need for iterative refinement, testing, and monitoring of AI tools across various settings, and enabling continuous user input for improvement.
- *I* represents *Inclusive communication*, focused on improving innovations that foster effective communication and decrease language and social barriers with and across diverse users and stakeholders.
- Finally, *T* signifies *Technical interoperability*. Participants highlighted the necessity of scalability and integration of AI models across heterogeneous infrastructures (e.g., data standards, electronic health record systems, and information system architectures), enabling compatibility across institutions and regions.

### Prototype

Each group developed, refined, and strengthened a set of three to four proposed solutions and requirements to advance inclusivity in health AI (*Prototype* stage). The proposed solutions addressed various stages of the AI lifecycle and targeted both structural and procedural challenges (Table 3).

**Table 3:** Summary of proposed solutions by challenge.

	A – Integrating lived experiences and stakeholder voices across the AI lifecycle	B – Designing data collection practices to promote fairness and prevent inequalities	C – Fostering regulatory frameworks to uphold human rights and promote inclusivity
**Focus area**	Operationalisation of ethical and regulatory frameworks; participatory development	Equity in data governance; education and incentives	Regulatory support; community empowerment
**Proposed solutions**	1. Develop a toolbox to support navigation of ethical and regulatory guidelines 2. Systematically involve diverse patient populations across AI lifecycle stages 3. Establish a European-level curriculum/training programme for patient representatives in AI	4. Improve transparency in data collection involving vulnerable populations 5. Embed data literacy in healthcare education 6. Create structural incentives (e.g., funding eligibility linked to data literacy) 7. Promote public and academic sector leadership in AI development	8. Establish a multi-stakeholder advisory panel on inclusive AI 9. Create a compliance support tool for inclusivity alignment 10. Collect and disseminate grassroots success stories to foster mutual learning
**Intended impact**	- Support practical adoption of existing guidelines, particularly among developers, to create more impactful and responsible AI applications - Promote sustained, meaningful stakeholder engagement - Build capacity among patient representatives	- Foster fairness and equity in data practices - Enhance critical data skills among professionals - Reduce industry dependency - Enable more inclusive AI innovation ecosystems	- Strengthen institutional guidance and accountability - Support implementation of inclusivity principles - Amplify local innovations and bottom-up engagement

Group A focused on facilitating the practical implementation of existing ethical and regulatory guidelines by proposing the development of a comprehensive toolbox that breaks down how these frameworks, ranging from laws to inclusive AI principles in healthcare, can be applied throughout the AI development process. Additionally, the group highlighted the importance of systematically involving diverse patient populations throughout the AI development process and suggested establishing a European-level learning programme or course to train and empower patient representatives in AI. This programme would aim to build the literacy, capacity, and confidence of patient representation to engage meaningfully and sustainably in AI design and governance processes. Another proposal involved organising bi-directional events, enabling patients and developers to gain a mutual understanding, fostering patient empowerment, and enhancing their agency in shaping AI solutions.

Group B proposed greater transparency and inclusiveness in data collection practices, particularly when involving vulnerable populations. They argued that data collection should become an explicit and mutual process between data collectors or domain experts (e.g., healthcare providers, researchers) and data subjects (e.g., patients, underserved groups), especially in encounters where such interactions are currently implicit or absent. The group advocated for embedding data literacy in healthcare education and establishing structural institutional incentives, such as requiring data literacy as a prerequisite for funding eligibility criteria within national funding agencies. Additionally, they highlighted the necessity of structural reform to reduce reliance on industry-led AI development by strengthening leadership in the public and academic sectors.

Group C emphasised the necessity of system change through far-reaching engagement with all the participatory stakeholders to foster inclusive AI development. They proposed the establishment of a multi-stakeholder advisory panel, composed of diverse stakeholder representatives, to be actively engaged throughout the development of regulatory frameworks, thereby ensuring that inclusivity principles are embedded across the AI lifecycle (focusing on the level of engagement). In parallel, they advocated for creating a compliance support tool integrating an interactive legal and ethical database with access to human legal expertise to assist developers and institutions in navigating regulatory and ethical requirements (focusing on the level of system change). Group C proposed systematically collecting and disseminating success stories from grassroots communities to support bottom-up engagement, which reflects diverse value priorities and local innovations (focusing on the level of incentives). By increasing policymakers’ awareness of these community-driven practices, the initiative would seek to stimulate responsive policy changes, thereby fostering a dynamic interaction between bottom-up initiatives and top-down governance in AI innovation.

Patient contributions on Day 2 served as a basis to develop the prototype solutions on Day 4 (Table 3). For example, in the discussion of Group A, it surfaced that underrepresented patients lack the participation and inclusion in health AI needed to secure their autonomy and accessibility; this directly motivated the proposed European-level training programme for patient representatives in AI. In Group B, the patient expert’s account of difficulties navigating the healthcare system led the group to prioritise data collection practices capable of revealing patient vulnerabilities and otherwise unseen or disempowered needs, a framing that informed the prototype emphasis on transparent, mutual data collection with vulnerable groups. In Group C, the persona and journey map highlighted how access to care is impeded when the system presumes patients can act proactively and navigate fragmented, efficiency-driven services; this shaped the proposed solution of collecting and disseminating grassroots success stories to foster mutual learning.

### Prioritise

At the *Prioritise* stage, participants identified and debated common themes and gaps for advancing inclusive health AI. The findings reported here are based on the subsequent analysis of these discussions (Table 4).

**Table 4:** Summary of shared priorities and gaps for advancing inclusive health AI.

Category	Description
**Shared Priorities**	- Development of support tools for guideline adoption and compliance - Mechanisms for meaningful inclusion of diverse voices - Integration of AI and data literacy into educational curricula - Design of incentive structures to promote inclusivity - Empowerment of marginalised communities - Systemic, value-driven change - Cross-boundary collaboration among disciplines, sectors, and stakeholders
**Gaps**	- Complexity and extended timelines of implementing inclusive, systemic solutions - Difficulty in operationalising ethical principles amid rapid technological change and innovation - Challenge of reconciling standardisation with value pluralism in diverse healthcare contexts - Fragmentation of disciplinary, lifecycle, and stakeholder efforts - Lack of proactive design for inclusivity (retrospective approaches dominate) - Limited and inconsistent stakeholder interaction and knowledge exchange - Insufficient patient involvement in innovation processes - Weak mechanisms for influencing policy change

Several overlapping themes emerged across these group discussions and the proposed solutions, reflecting a shared understanding of the systemic changes required to achieve inclusive health AI. These included the development of support tools to assist in guideline adoption and compliance, mechanisms for the meaningful inclusion of diverse voices, and the integration of AI and data literacy into educational curricula. Other recurring themes involved designing incentive structures to encourage inclusive practices, such as linking funding to specific actions that promote data literacy and transparency. These incentives were seen as essential for empowering marginalised communities and driving broader systemic, value-driven change. The participants also recognised the fragmented nature of current efforts, particularly siloed approaches across disciplines, the uneven attention to different stages of the AI lifecycle, and the lack of integrated stakeholder engagement mechanisms. This underscored the importance of cross-boundary collaboration among disciplines, sectors, and stakeholders.

Despite the promising nature of these proposals, participants acknowledged several persistent gaps that must be addressed to advance inclusive health AI. One such gap is the inherent complexity and extended timelines involved in implementing inclusive solutions, particularly those that require systemic change, such as embedding participatory mechanisms across the AI lifecycle, reforming data governance structures, or aligning regulatory frameworks with human rights principles. These efforts often demand sustained stakeholder engagement, cross-sector coordination, and iterative testing, which can be resource-intensive and slow-moving.

Another gap raised by participants was safeguarding ethical principles during rapid technological change. While many stakeholders invoke ethical principles, such as fairness, transparency, or accountability, participants noted that these principles are often inconsistently applied or lack operational clarity in practice. The challenge lies not in the absence of ethical discourse, but in translating ethical principles into actionable, context-sensitive mechanisms that can keep pace with technological innovation. Participants emphasised the need for tools, training, and governance structures that support ethical alignment throughout the AI lifecycle, especially in high-stakes domains like healthcare.

A third gap involved reconciling standardisation with value pluralism. Participants noted that many AI systems in healthcare are designed for scalability and interoperability, which often necessitates standardised protocols and data formats. However, healthcare contexts are inherently diverse: patients, communities, and institutions may hold differing values, priorities, and definitions of equitable care. The challenge is not that unified systems are incompatible with diverse values, but that without deliberate design, standardisation can obscure or override context-specific needs. Participants emphasised the importance of embedding mechanisms for local adaptation, stakeholder input, and value-sensitive design to ensure that AI systems remain responsive to pluralistic healthcare environments.

Furthermore, participants emphasised the need for systems that are proactively designed to be inclusive from the beginning rather than retrospectively. They stressed the importance of sustained knowledge exchange and interaction among stakeholders, greater patient involvement in shaping innovation trajectories, and more effective mechanisms for influencing policy.

### PREFER-IT framework consolidation

**Fig 4:**
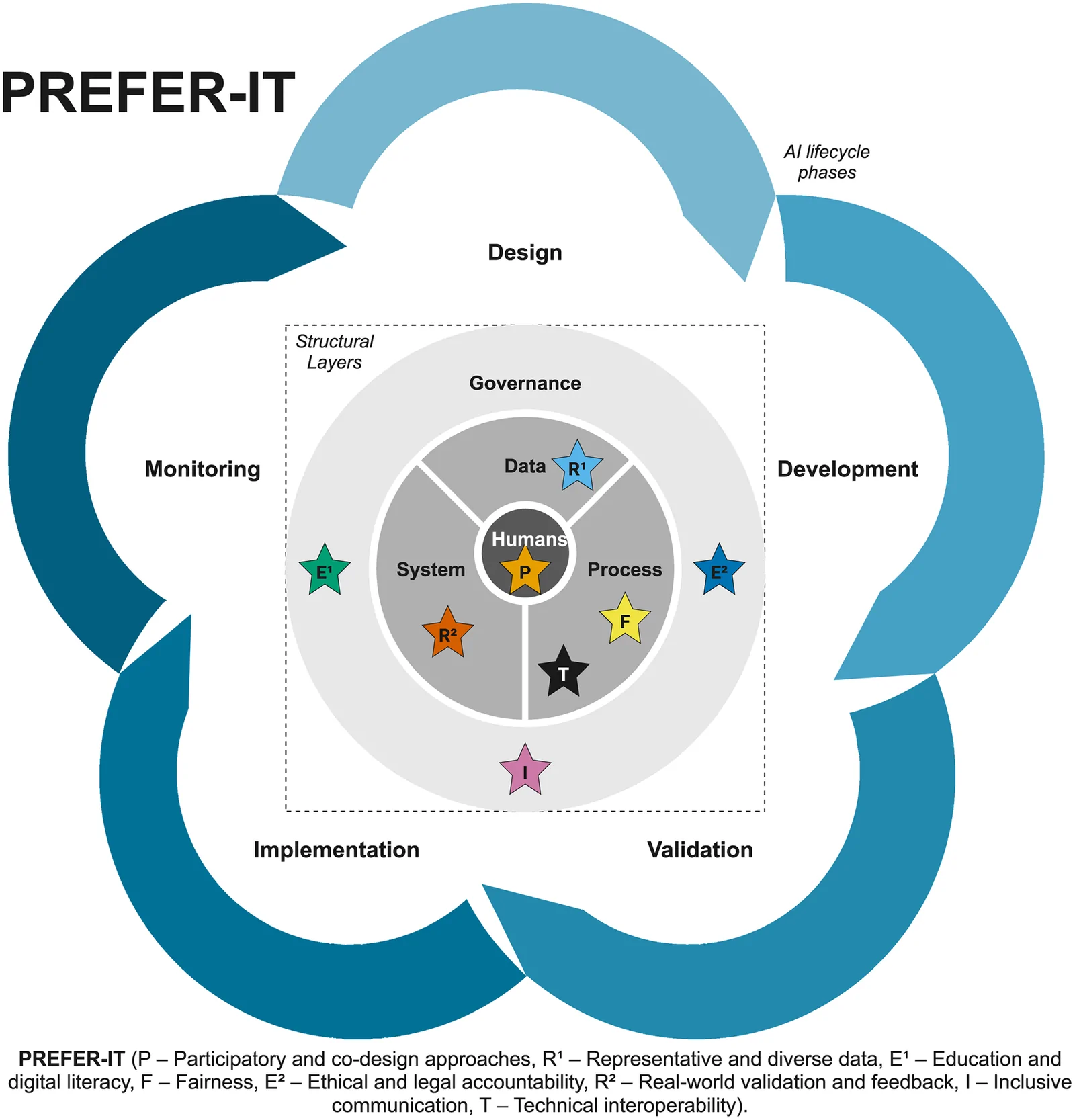
Implementation of the PREFER-IT framework across structural layers and the AI lifecycle.

To strengthen the practical value of PREFER-IT, we mapped it onto a dual-axis model (Fig 4), combining Zowghi and da Rimini’s structural layers of AI (humans, data, process, system, governance) [30] with the phases of the AI lifecycle (design, development, validation, implementation, monitoring) [2]. The *humans* pillar emphasises the centrality of stakeholder involvement and diverse representation. *Data* focuses on equitable and unbiased practices in the collection, labelling, modelling, and use. *Process* covers the activities across the lifecycle that lead to the delivery of an AI system. The *system* refers to the AI system itself, which must be evaluated, tested, and monitored in context. Finally, the *governance* pillar comprises the structures, processes, and regulatory frameworks that ensure compliance and ethical alignment. This approach situates PREFER-IT elements as requirements that can orient and sustain inclusivity across different dimensions of health AI.

Importantly, the boundaries of PREFER-IT within this dual axis are not meant to be static, but are intentionally flexible. This flexibility reflects the reality that many requirements intersect across different AI structural layers and lifecycle phases, underscoring the interdependent and systemic nature of inclusivity in AI.

For simplification purposes, in Fig 4 each component of the PREFER-IT framework aligns with specific structural layers and phases of the health AI lifecycle. For instance, Participatory and co-design approaches (P) are essential throughout all stages of the AI lifecycle and must actively involve all stakeholders (labelled as “humans”). Representative and diverse data (R) plays a crucial role within the data layer, particularly during the design phase. Elements such as Education and digital literacy (E), Ethical and legal accountability (E), and Inclusive communication (I) are fundamental governance considerations that span the entire AI lifecycle. Meanwhile, Fairness (F) and Technical interoperability (T) are core to the process layer and should be prioritised during development and validation. Finally, Real-world validation and feedback (R) are vital in any AI system, ensuring its adaptation to evolving socio-technical contexts, and should be considered during the validation, implementation, and monitoring stages.

**Fig 5:**
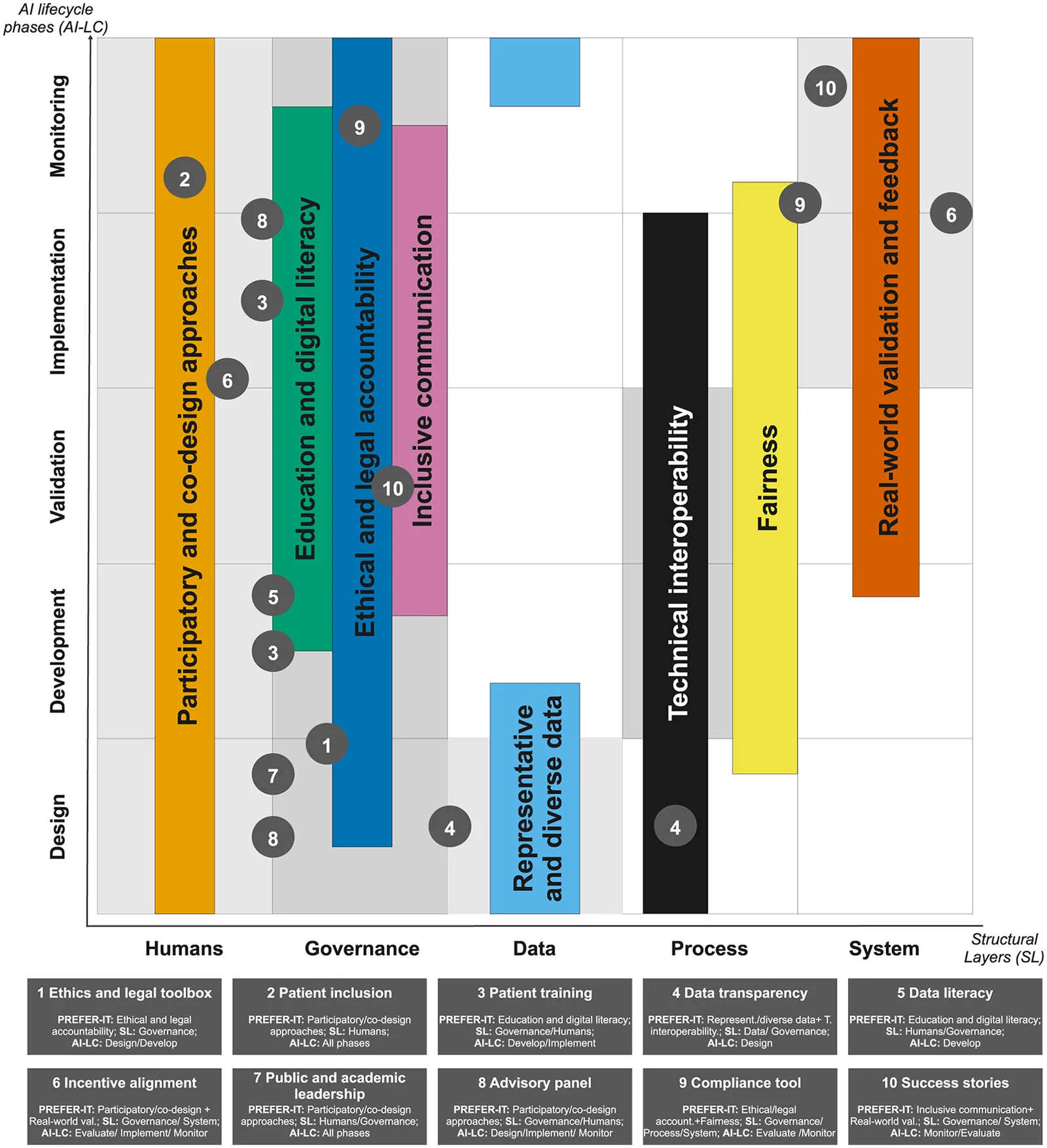
Operationalising PREFER-IT. Mapping of the prototype solutions co-created during the workshop (from Table 3) across the dual-axis of PREFER-IT (AI structural layers and lifecycle stages).

To demonstrate a proof of concept, we mapped the representative prototype solutions developed during the in-person workshop (from Table 3) onto the dual-axis of PREFER-IT (Fig 5). This exercise highlights how the framework can be applied in practice with concrete solutions across the AI ecosystem.

A single solution can justifiably be placed in different locations depending on the perspective of the team or stakeholder group conducting the mapping. Rather than undermining the map’s purpose, this adaptability reinforces its value as a practical tool for navigating complexity. The value of this exercise is not to produce a definitive categorisation, but to use PREFER-IT as a guiding framework that ensures inclusivity remains a core consideration throughout the design, development, validation, implementation, monitoring, and governance of AI in healthcare.

## Discussion

This paper presents PREFER-IT, a set of co-created foundational requirements developed through a transdisciplinary workshop to provide an operational framework for realising inclusivity throughout the AI lifecycle and governance in healthcare. By engaging a diverse group of stakeholders in a structured co-creation process, we translated lived experiences and expert insights into actionable requirements for inclusive health AI.

The workshop combined a preparatory PPI meeting and survey, a consensus-building exercise, and design thinking methods, including patient personas and journey mapping with the active involvement of patient experts. This structured process progressively moved from the broad challenge of inclusive health AI to a set of concrete, experience-informed solutions and requirements, eventually culminating in the development of the PREFER-IT framework.

While existing frameworks often address fairness, trustworthiness, or ethics in health AI [2,9,18,25,44–47], they generally remain conceptual or focus on technical dimensions. A unified, actionable framework for AI inclusivity, particularly within healthcare, remains absent, at least to our knowledge. PREFER-IT is a first step to respond to this gap by offering both a conceptual and applied foundation to guide the inclusive design, development, validation, implementation, monitoring, and governance of health AI.

The co-creation process involved a small number of participants, providing in-depth, exploratory knowledge exchange that generated valuable insights. The diversity of expertise and richness of perspectives provide a robust qualitative foundation for further empirical investigation. The co-created solutions, grounded in patient voices and lived experiences, particularly emphasised the human and governance dimensions of AI, reflecting the workshop’s interdisciplinary composition across social sciences, ethics, and health policy. However, the framework remains adaptable to technical domains and can guide integration at the data, process, and system levels of health AI. While the discussions were primarily situated within a European context, this framing offers a strong basis for extending and validating the framework in other settings. A possible next step could be to test and adapt the framework across diverse contexts to capture a broader range of epistemic perspectives and societal concerns, ensuring its global relevance and applicability.

The workshop also revealed the importance of authentic participation. While patient involvement enriched the framework, it also highlighted risks of tokenism. Similar tensions have been observed in participatory AI design more broadly, where researchers and practitioners often struggle to reconcile participatory ambitions with practical constraints such as time, resources, and institutional norms [48]. To navigate these challenges, teams frequently rely on proxies (like stand-ins for stakeholders) that may inadvertently reinforce existing power asymmetries rather than dismantle them [48]. Institutional epistemic trust in AI-inclusive healthcare depends on how patients’ values shape the system and how power and commercial interests are distributed around it [3]. When participation is only nominal, that relational basis for trust is weakened. This underscores the need for co-creation to extend beyond isolated workshops toward sustained, reflexive engagement mechanisms that distribute decision-making authority and epistemic legitimacy. Without such safeguards, inclusion risks being symbolic rather than transformative. The traceable influence of patient input on the group problem statements and prototype solutions (see Results) offers some evidence that participation here moved beyond symbolic presence; nonetheless, a single workshop cannot substitute for the sustained, reflexive engagement we argue is necessary.

Participants also identified several persistent gaps for realising inclusive health AI. Rather than obstacles, these can be reframed as priority areas where PREFER-IT should be actively applied or advocated. For instance, the call for support tools to facilitate guideline adoption and compliance highlights the need for stronger public engagement in shaping and implementing AI regulation, as illustrated by the Algorithm Register of the Dutch government [49]. At the policy level, incorporating PREFER-IT principles into funding criteria, regulatory assessments, and institutional governance could help move inclusion from aspiration to routine practice. This approach would shape AI health inclusivity in *what* is developed, *how* it is developed, *who* develops it, and *how* it is evaluated and refined over time.

Likewise, the demand for educational and capacity-building initiatives, such as patient training programmes or education tools, aligns with ongoing policy efforts, such as those outlined in the AI Act [50], to support sustained implementation of PREFER-IT. Capacity-building should also target AI developers and HCP to bridge disciplinary gaps. As AI researchers often lack health expertise, collaboration with clinicians and patients is crucial for understanding the clinical context and translating patient needs into technical design requirements.

The more systemic concerns, including implementation complexity, safeguarding ethics, and balancing diverse values, highlight that PREFER-IT should not be approached as a simple checklist. Instead, it needs to be an adaptable infrastructure spanning the entire AI lifecycle and its various structural layers. The core challenge lies not in the absence of ethical discourse but in translating principles into actionable, context-sensitive mechanisms that can keep pace with technological innovation. Viewing inclusivity as a behavioural process that requires capability, opportunity, and motivation could strengthen the framework’s practical applicability and promote organisational cultures that sustain inclusive AI development [51].

Adopting PREFER-IT should begin at project inception and not at the point of evaluation. The dual-axis map (Fig 4) can serve as a planning canvas during the design phase: for each intersection of structural layer and lifecycle phase, the team can ask which PREFER-IT requirements are relevant, what concrete actions they imply, and who is accountable for them. Assigning ownership of each element to a named role (e.g., a team member responsible for sustained Participatory and co-design engagement (P)) counters the diffusion of responsibility that often leaves inclusivity unattended. Because the boundaries of PREFER-IT are flexible, the map should be revisited at each AI lifecycle transition, allowing requirements to be reweighted as the project and its stakeholders evolve.

Applied this way, PREFER-IT also gives teams a structure for navigating the systemic tensions our workshop surfaced, illustrated by three of the gaps participants identified. First, standardisation can conflict with value pluralism. An interoperable social determinant of health value set may encode housing and food insecurity yet contain no coded concept for residence-status-driven care avoidance, a determinant central to the care of undocumented migrants. The variable then collapses to an unmapped field and is silently lost at the mapping step. The framework prompts the team to document that the interoperable data standard (T) cannot represent this relevant determinant of health (R), record why they nonetheless proceed, and specify a local adaptation mechanism, so the trade-off remains visible and revisable.

Second, ethical principles risk being invoked without being operationalised. Fairness (F) or accountability (E) may be asserted in a proposal, yet never translated into a concrete, testable mechanism, exerting no real constraint as the AI system is built. The framework counters this by tying each principle to a specific step across the AI lifecycle, converting ethical discourse into verifiable commitments.

Third, meaningful inclusion risks collapsing into tokenism. When patients are consulted only after research questions and evaluation metrics are fixed, their input cannot reshape the study. The framework mitigates this by requiring that participation (P) is planned, resourced, and budgeted from the proposal stage, with patient representatives helping to define the questions and metrics themselves.

At the institutional level, PREFER-IT could be embedded within governance mechanisms that already shape AI projects, avoiding the creation of parallel and easily bypassed procedures. Its requirements could be incorporated into research ethics review, data protection impact assessments, clinical validation gates, and procurement and funding criteria, so that inclusivity is examined at the same checkpoints as safety and efficacy. Linking specific PREFER-IT actions to funding eligibility (as workshop participants suggested), or to internal AI governance sign-off, would help convert the framework from voluntary aspiration into routine practice, consistent with capacity-building obligations such as those articulated in the AI Act [50] and with public-facing accountability instruments such as the Algorithm Register of the Dutch government [49].

Looking ahead, the next steps involve translating PREFER-IT from a co-created framework into a living infrastructure for inclusive health AI. This will require ongoing engagement with diverse stakeholders, including patients with intersectional vulnerabilities, regulators, and insurers. It also involves iterative testing in real-world projects, systematic evaluation across healthcare contexts, and continuous refinement based on user and patient feedback. Through this operationalisation, PREFER-IT aims to advance AI in healthcare that is not only effective but also equitable, ethical, legal, and socially responsible [52].

The co-creation process highlighted that realising inclusivity in health AI requires more than just design features, depending on systemic enablers across multiple levels. In line with Wang and Blok’s multi-level framework [25], PREFER-IT can be operationalised at the micro-level (individual AI issues), meso-level (organisational and systemic issues), and macro-level (philosophical issues). For example, policymakers can mandate transparency and equity audits; developers can integrate inclusion-by-design and participatory testing; researchers and clinicians can promote representative study designs; and patients and communities can co-define research priorities and evaluation metrics. The PREFER-IT framework could provide a basis for coordinating these actions across roles and governance levels.

Finally, our research suggests a broader reframing: from an *innovation-first* logic to an *inclusive-by-design* paradigm. This shift positions inclusivity as a constitutive element of health AI, embedded not only in outcomes but also in processes, governance structures, and power relations. By doing so, PREFER-IT helps address epistemic hierarchies in knowledge generation, challenging the dominance of quantitative and technical evidence and elevating the value of qualitative insights, experiential knowledge, and patient voices in shaping AI development.

Through the development of PREFER-IT, we offer both a conceptual contribution and a practical resource to support stakeholders in realising inclusivity throughout the AI lifecycle in healthcare. The framework builds on, but also extends beyond, existing frameworks by positioning inclusion not as a peripheral ethical concern but as a core design principle, rooted in equity, stakeholder engagement, and governance accountability.

Crucially, this process revealed that advancing inclusion in health AI is not merely a matter of technological or design choices. It requires reconfiguring institutional logics, incentive structures, and policy environments. The PREFER-IT framework captures these multi-level dynamics and provides a basis for coordinated action. By explicitly incorporating the roles of patients and communities, it helps bridge the gap between AI development and implementation and the lived realities of those most affected by it.

Ultimately, this process challenges dominant epistemic practices and paradigms in health innovation, which often prioritise efficiency and technical advancement over equity and participation. Co-creation, as adopted here, is not merely a methodological technique but a stance that recognises the value of inclusion. It shifts the focus from innovation *for* to innovation *with* communities.

## Supporting information

S1 Fig Field of expertise and seniority of workshop contributors by category. (PDF)

S2 Fig Relevant socio-ethical issues of inclusive health AI, adapted from Wang and Blok’s multi-level framework. Adapted from: [25]. This article is distributed under the terms of the Creative Commons Attribution 4.0 License (https://creativecommons.org/licenses/by/4.0/), which permits any use, reproduction, and distribution of the work without further permission, provided the original work is properly attributed as specified on the SAGE Open Access page (https://us.sagepub.com/en-us/nam/open-access-at-sage).
(PDF)

S3 Fig Prepared templates. (PDF)

S4 Fig Graphical abstract. The icons in the graphical abstract were adapted by the authors from clipart obtained from OpenClipArt (https://openclipart.org), released under the Creative Commons CC0 1.0 Universal Public Domain Dedication (https://creativecommons.org/publicdomain/zero/1.0/).
(PDF)

S1 Table Results from Polis. (PDF)

S2 Table Complete problem statements for each challenge. (PDF)
