# Supplementary material for: PREFER-IT: A transdisciplinary co-created framework to realise inclusive health AI
Source: PLOS Digit Health. 2026 Sep 23;5(9):e0001096. doi: 10.1371/journal.pdig.0001096 (PMC13600515; doi:10.1371/journal.pdig.0001096)
Supplement: Supplementary file 1 [file pdig.0001096.s001.pdf]

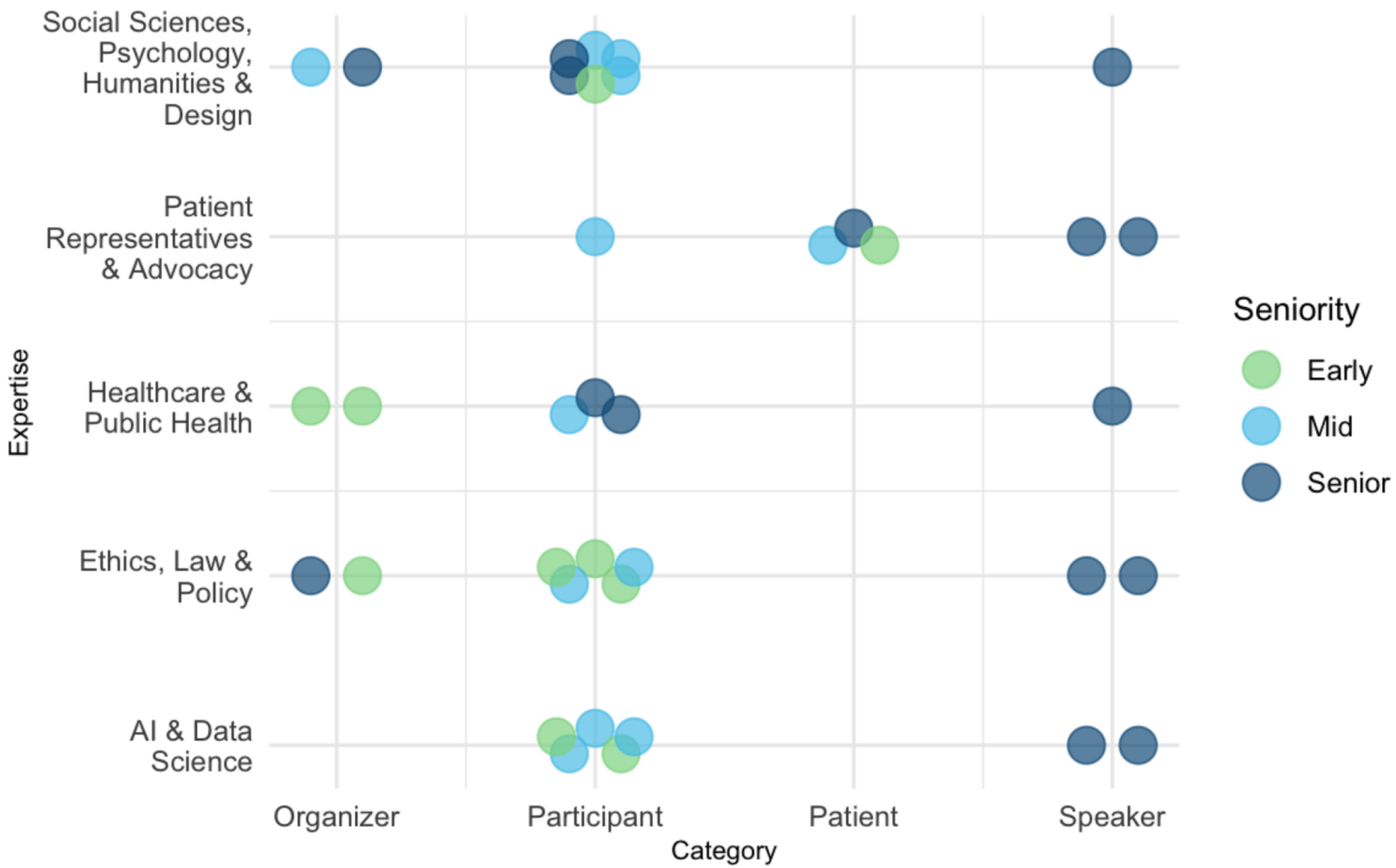

S1 Fig. Field of expertise and seniority of workshop contributors by category. Distribution of experts involved in the workshop "Inclusive Medical AI: Exploring Diverse Perspectives" according to category, field of expertise, and seniority.
