# Supplementary material for: PREFER-IT: A transdisciplinary co-created framework to realise inclusive health AI
Source: PLOS Digit Health. 2026 Sep 23;5(9):e0001096. doi: 10.1371/journal.pdig.0001096 (PMC13600515; doi:10.1371/journal.pdig.0001096)
Supplement: Supplementary file 2 [file pdig.0001096.s002.pdf]

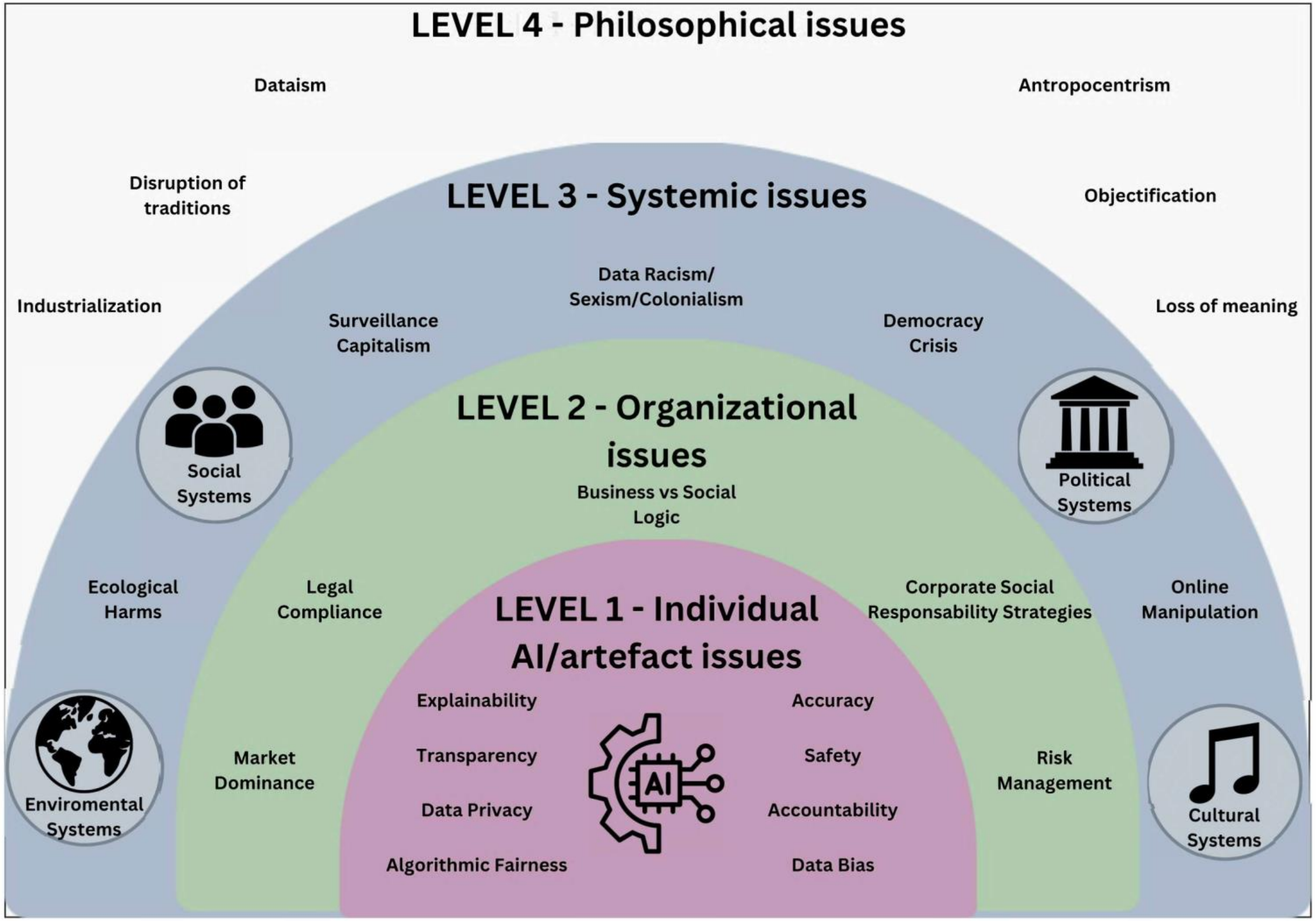

S2 Fig. Relevant socio-ethical issues of inclusive health AI, adapted from Wang and Blok's multi-level framework. The framework was used to map the identified challenges of inclusive health AI and their corresponding requirements across different levels.
