# Supplementary material for: PREFER-IT: A transdisciplinary co-created framework to realise inclusive health AI
Source: PLOS Digit Health. 2026 Sep 23;5(9):e0001096. doi: 10.1371/journal.pdig.0001096 (PMC13600515; doi:10.1371/journal.pdig.0001096)
Supplement: Supplementary file 3 [file pdig.0001096.s003.pdf]

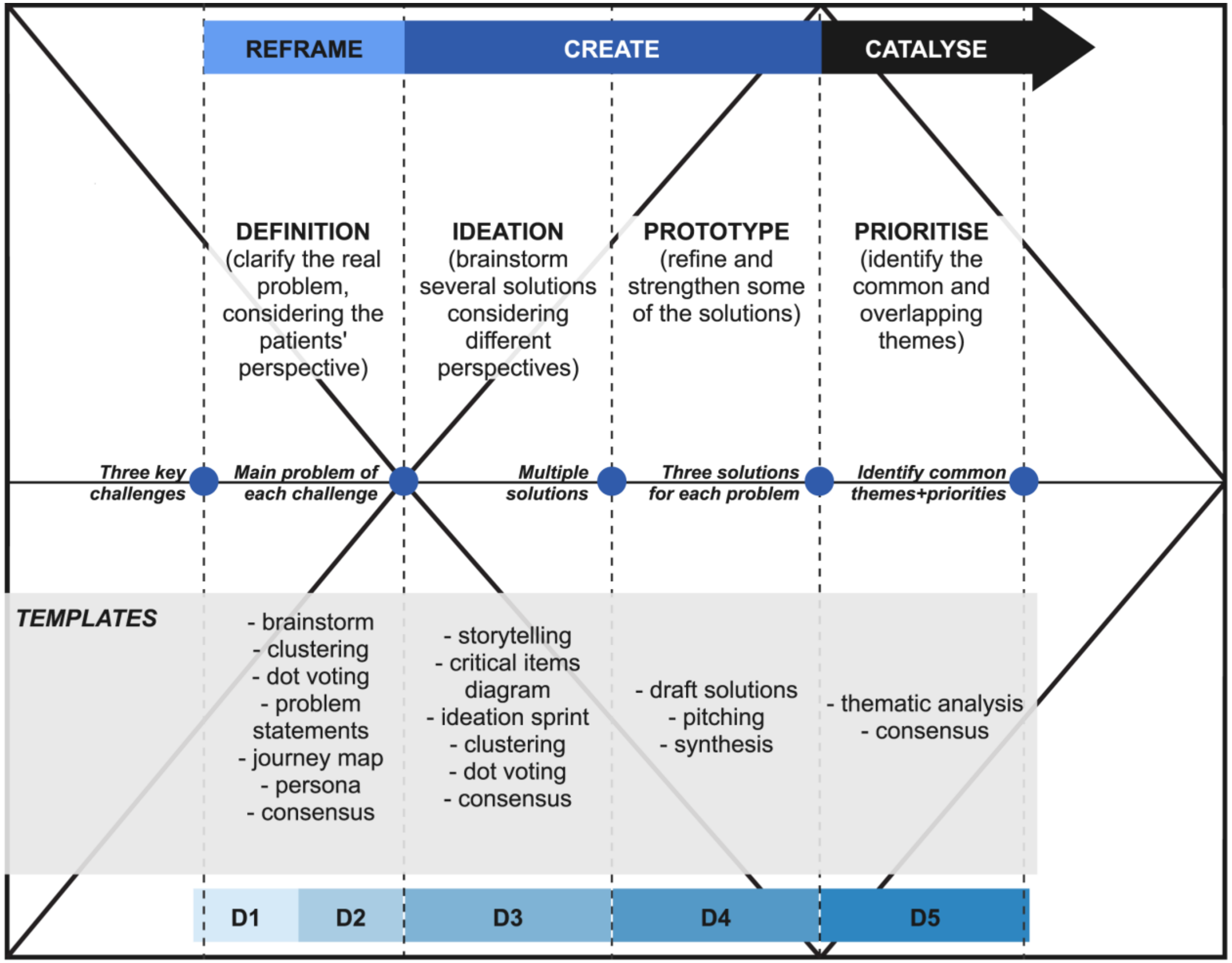

S3 Fig. Prepared templates. Templates prepared for each day of the workshop, based on the double diamond model and the innovation and systemic design frameworks [36–38].
