# Supplementary material for: PREFER-IT: A transdisciplinary co-created framework to realise inclusive health AI
Source: PLOS Digit Health. 2026 Sep 23;5(9):e0001096. doi: 10.1371/journal.pdig.0001096 (PMC13600515; doi:10.1371/journal.pdig.0001096)
Supplement: Supplementary file 5 [file pdig.0001096.s005.pdf]

S1 Table. Results from Polis.

ID statement	Statement	Overall (N=27)					Group A (N=10)					Group B (N=4)					Group C (N=5)					Group D (N=8)				
		Total votes	Agree	Disagree	Pass	No vote	Total votes	Agree	Disagree	Pass	No vote	Total votes	Agree	Disagree	Pass	No vote	Total votes	Agree	Disagree	Pass	No vote	Total votes	Agree	Disagree	Pass	No vote
		N	N (%)	N (%)	N (%)	N	N	N (%)	N (%)	N (%)	N	N	N (%)	N (%)	N (%)	N	N	N (%)	N (%)	N (%)	N	N	N (%)	N (%)	N (%)	N
0	Developing methods to minimize biases in AI systems.	27	18<br>66,7%	4<br>14,8%	5<br>18,5%	0	10	5<br>50,0%	2<br>20,0%	3<br>30,0%	0	4	4<br>100,0%	0<br>0,0%	0<br>0,0%	0	5	5<br>100,0%	0<br>0,0%	0<br>0,0%	0	8	4<br>50,0%	2<br>25,0%	2<br>25,0%	0
1	Understanding the effects of bias on AI outputs, such as unfair recommendations.	27	15<br>55,6%	3<br>11,1%	9<br>33,3%	0	10	2<br>20,0%	3<br>30,0%	5<br>50,0%	0	4	4<br>100,0%	0<br>0,0%	0<br>0,0%	0	5	5<br>100,0%	0<br>0,0%	0<br>0,0%	0	8	4<br>50,0%	0<br>0,0%	4<br>50,0%	0
2	Including diverse stakeholder voices throughout the AI lifecycle.	26	21<br>80,8%	1<br>3,8%	4<br>15,4%	1	9	7<br>77,8%	0<br>0,0%	2<br>22,2%	1	4	3<br>75,0%	1<br>25,0%	0<br>0,0%	0	5	5<br>100,0%	0<br>0,0%	0<br>0,0%	0	8	6<br>75,0%	0<br>0,0%	2<br>25,0%	0
3	Including the lived experiences of diverse groups in AI design and implementation.	27	22<br>81,5%	0<br>0,0%	5<br>18,5%	0	10	8<br>80,0%	0<br>0,0%	2<br>20,0%	0	4	4<br>100,0%	0<br>0,0%	0<br>0,0%	0	5	5<br>100,0%	0<br>0,0%	0<br>0,0%	0	8	5<br>62,5%	0<br>0,0%	3<br>37,5%	0
4	Developing regulatory frameworks that address inclusivity in health AI.	26	15<br>57,7%	4<br>15,4%	7<br>26,9%	1	9	4<br>44,4%	3<br>33,3%	2<br>22,2%	1	4	1<br>25,0%	1<br>25,0%	2<br>50,0%	0	5	5<br>100,0%	0<br>0,0%	0<br>0,0%	0	8	5<br>62,5%	0<br>0,0%	3<br>37,5%	0
5	Creating regulatory frameworks interpretable for all stakeholders.	27	10<br>37,0%	9<br>33,3%	8<br>29,6%	0	10	0<br>0,0%	6<br>60,0%	4<br>40,0%	0	4	0<br>0,0%	2<br>50,0%	2<br>50,0%	0	5	5<br>100,0%	0<br>0,0%	0<br>0,0%	0	8	5<br>62,5%	1<br>12,5%	2<br>25,0%	0
6	Ensuring training datasets represent diverse groups.	27	18<br>66,7%	3<br>11,1%	6<br>22,2%	0	10	5<br>50,0%	2<br>20,0%	3<br>30,0%	0	4	2<br>50,0%	1<br>25,0%	1<br>25,0%	0	5	5<br>100,0%	0<br>0,0%	0<br>0,0%	0	8	6<br>75,0%	0<br>0,0%	2<br>25,0%	0
7	Assessing whether current data collection practices exacerbate inequalities.	27	20<br>74,1%	3<br>11,1%	4<br>14,8%	0	10	5<br>50,0%	3<br>30,0%	2<br>20,0%	0	4	4<br>100,0%	0<br>0,0%	0<br>0,0%	0	5	5<br>100,0%	0<br>0,0%	0<br>0,0%	0	8	6<br>75,0%	0<br>0,0%	2<br>25,0%	0

8	Investigating the societal impact of AI systems on diverse groups.	27	17	5	5	0	10	4	4	2	0	4	4	0	0	0	5	5	0	0	0	8	4	1	3	0
			63,0%	18,5%	18,5%			40,0%	40,0%	20,0%			100,0%	0,0%	0,0%			100,0%	0,0%	0,0%			50,0%	12,5%	37,5%	
9	Continuous evaluation of AI across diverse groups.	26	13	4	9	1	9	4	3	2	1	4	1	1	2	0	5	5	0	0	0	8	3	0	5	0
			50,0%	15,4%	34,6%			44,4%	33,3%	22,2%			25,0%	25,0%	50,0%			100,0%	0,0%	0,0%			37,5%	0,0%	62,5%	
10	Identifying how health AI affects human rights of diverse groups.	27	10	9	8	0	10	0	7	3	0	4	1	1	2	0	5	5	0	0	0	8	4	1	3	0
			37,0%	33,3%	29,6%			0,0%	70,0%	30,0%			25,0%	25,0%	50,0%			100,0%	0,0%	0,0%			50,0%	12,5%	37,5%	
11	Identifying how health AI affects public and personal values of diverse groups.	27	15	3	9	0	10	4	3	3	0	4	4	0	0	0	5	4	0	1	0	8	3	0	5	0
			55,6%	11,1%	33,3%			40,0%	30,0%	30,0%			100,0%	0,0%	0,0%			80,0%	0,0%	20,0%			37,5%	0,0%	62,5%	
12	Incorporating AI advancements in healthcare context.	26	10	9	7	1	9	0	6	3	1	4	4	0	0	0	5	4	0	1	0	8	2	3	3	0
			38,5%	34,6%	26,9%			0,0%	66,7%	33,3%			100,0%	0,0%	0,0%			80,0%	0,0%	20,0%			25,0%	37,5%	37,5%	
13	Monitoring and detecting problems and risks in AI systems before their release.	27	17	4	6	0	10	4	3	3	0	4	3	0	1	0	5	4	1	0	0	8	6	0	2	0
			63,0%	14,8%	22,2%			40,0%	30,0%	30,0%			75,0%	0,0%	25,0%			80,0%	20,0%	0,0%			75,0%	0,0%	25,0%	
14	Collaborating between different scientific disciplines.	27	14	4	9	0	10	2	3	5	0	4	4	0	0	0	5	5	0	0	0	8	3	1	4	0
			51,9%	14,8%	33,3%			20,0%	30,0%	50,0%			100,0%	0,0%	0,0%			100,0%	0,0%	0,0%			37,5%	12,5%	50,0%	
15	Using a systems approach.	25	7	4	14	2	8	2	3	3	2	4	4	0	0	0	5	0	1	4	0	8	1	0	7	0
			28,0%	16,0%	56,0%			25,0%	37,5%	37,5%			100,0%	0,0%	0,0%			0,0%	20,0%	80,0%			12,5%	0,0%	87,5%	
16	As a matter of principle, I don't think an AI tool should be used in the process of determining which topics are most pertinent.	25	4	16	5	2	9	1	6	2	1	4	0	3	1	0	4	1	3	0	1	8	2	4	2	0
			16,0%	64,0%	20,0%			11,1%	66,7%	22,2%			0,0%	75,0%	25,0%			25,0%	75,0%	0,0%			25,0%	50,0%	25,0%	
17	Inventory and development of methods to include people in process of AI development and implementation	22	12	3	7	5	8	4	1	3	2	4	1	2	1	0	2	1	0	1	3	8	6	0	2	0
			54,5%	13,6%	31,8%			50,0%	12,5%	37,5%			25,0%	50,0%	25,0%			50,0%	0,0%	50,0%			75,0%	0,0%	25,0%	

18	Address AI inclusivity in different stages of the AI lifecycle	22	16	1	5	5	8	6	1	1	2	4	3	0	1	0	2	2	0	0	3	8	5	0	3	0
			72,7%	4,5%	22,7%			75,0%	12,5%	12,5%			75,0%	0,0%	25,0%			100,0%	0,0%	0,0%			62,5%	0,0%	37,5%	
19	Address AI inclusivity at different ‘system’ levels (micro, meso, macro)	22	16	1	5	5	8	6	1	1	2	4	3	0	1	0	2	1	0	1	3	8	6	0	2	0
			72,7%	4,5%	22,7%			75,0%	12,5%	12,5%			75,0%	0,0%	25,0%			50,0%	0,0%	50,0%			75,0%	0,0%	25,0%	
20	Develop methods/frameworks to promote inclusivity for health experts working with AI to increase safe adoption (e.g. AI literacy)	20	12	4	4	7	8	5	1	2	2	4	1	2	1	0	2	1	0	1	3	6	5	1	0	2
			60,0%	20,0%	20,0%			62,5%	12,5%	25,0%			25,0%	50,0%	25,0%			50,0%	0,0%	50,0%			83,3%	16,7%	0,0%	
21	Make sure AI is at the service of patients	19	9	3	7	8	7	3	2	2	3	4	3	0	1	0	2	2	0	0	3	6	1	1	4	2
			47,4%	15,8%	36,8%			42,9%	28,6%	28,6%			75,0%	0,0%	25,0%			100,0%	0,0%	0,0%			16,7%	16,7%	66,7%	
22	When developing and deploying healthcare AI, patients' rights should be taken into account.	6	4	1	1	21	1	0	1	0	9	0	0	0	0	4	1	1	0	0	4	4	3	0	1	4
			66,7%	16,7%	16,7%			0,0%	100,0%	0,0%			NA	NA	NA			100,0%	0,0%	0,0%			75,0%	0,0%	25,0%	

Legend: The statements highlighted in grey represent those with the highest levels of agreement (defined as having over 66% of participants selecting “Agree” across the entire sample). Participants were asked to vote on the statements, considering what they felt were the most significant challenges we should focus on during the five-day in-person workshop. This means that when participants choose “Disagree” or “Pass”, it is not necessarily because they disagree or are unsure about the statement, but because they felt it was not as big a priority to focus on the workshop as other statements. Among the 27 participants, four distinct opinion groups emerged in Polis. These groups were defined by two criteria: first, members within each group exhibited similar voting patterns across multiple statements; second, their voting behaviour was consistently different from that of participants in the other groups. Polis uses the k-means clustering algorithm to group participants into clusters and the silhouette coefficient to select an optimal number of clusters.
