# Supplementary material for: PREFER-IT: A transdisciplinary co-created framework to realise inclusive health AI
Source: PLOS Digit Health. 2026 Sep 23;5(9):e0001096. doi: 10.1371/journal.pdig.0001096 (PMC13600515; doi:10.1371/journal.pdig.0001096)
Supplement: Supplementary file 6 [file pdig.0001096.s006.pdf]

S2 Table. Complete problem statements for each challenge

	**A – Integrating lived experiences and stakeholder voices across the AI lifecycle**	**B – Designing data collection practices to promote fairness and prevent inequalities**	**C – Fostering regulatory frameworks to uphold human rights and promote inclusivity**
**Map the problems**	Three clusters:<br>1. Power; 2. Frameworks; 3. Representation	Four clusters:<br>1. Need/incentives for data; 2. Society;<br>3. Parameter conditions; 4. Processing	Eight clusters:<br>1. Semantics; 2. Trust and transparency;<br>3. Multilevel/systems thinking; 4. Power structure;<br>5. System development; 6. Policy; 7. Human rights;<br>8. Morality
**Problem statement**	1. We assume that for **business/healthcare companies,** there is a **lack of interactions with patients** to create **personalised solutions to** realise inclusive health AI.<br>2. We assume that for **underrepresented patients,** there is a **lack of participation and inclusion** to ensure **autonomy and accessibility** to realise inclusive health AI.<br>3. We assume that for **AI researchers,** there is a **difficulty in identifying patients' needs, knowledge, values and emotions** to successfully **categorise these** to realise inclusive health AI.	1. We assume that for **healthcare users,** there is a **lack of process, protocol or prerequisite of co-creation** to decide/determine which **data will be collected t**o realise inclusive health AI.<br>2. We assume that for **hidden & difficult-to-reach people, such as groups with low SES or intellectual disabilities**, there is a **lack of comprehensive insights into broader determinants of health**, requiring **alternative approaches to identify relevant data, determine how to collect and process it, and engage these populations meaningfully in research** to realise inclusive health AI.<br>3. We assume that for **AI developers,** there is a **lack of decisional autonomy/agency** to prioritise **appropriate/purposeful data collection** to realise inclusive health AI.	1. We assume that for **HCP, policy makers (and patients),** there is an **economic pressure as efficiency drivers** needing **stronger oversight** to realise inclusive health AI.<br>2. We assume that for **AI developers & regulatory bodies, there** is **uncertainty about how to comply with regulations,** needing **practical/actionable guidelines or tools** to realise inclusive health AI.<br>3. We assume that for **developers of regulatory frameworks,** there is a **limited and biased view of the levels and relevant voices** needing **a more creative and exhaustive stakeholder analysis** to realise inclusive health AI.
**Stakeholder map**	1. Business, developers, lobby organisations, HCP, patients, government, ethicists, research/academia<br>2. Business, lobby, ethicists, academia, HCP, government, developers, patients<br>3. Research/academia, business, developers, HCP, patients, ethicists, lobby, government	1. Funding agency, HCP, researchers, developers, managers of departments in hospitals, department of disciplinary guidelines, policy makers, technology providers, patients<br>2. Funding agency, community, citizens (non-patients), policy makers, HCP, patients<br>3. Funding agency, developer, patients (source of data), legislators	1. Partnership of health specialists, hospital board, policy makers (European Union, health ministry, regional and municipal levels), patients, and citizens (society)<br>2. AI developers, researchers/in-house developers, regulatory bodies, ICT departments, investors, private companies, patients, clinicians, multidisciplinary consultation, head of clinic, specialist partnership<br>3. Policy makers, researchers, AI developers, patients' organisation/representatives, private companies and the pharmaceutical industry, HCP

Legend: Complete problem statements and stakeholder maps for each challenge. The three statements per challenge correspond to distinct actors identified during the *Definition* stage; statements are formatted as design-thinking point-of-view statements. The stakeholder map lists, for each problem statement, the actors arranged by their capacity to influence the problem. AI – artificial intelligence; HCP – healthcare professional; SES – socioeconomic status; ICT – information and communications technology.
