# Supplementary material for: PREFER-IT: A transdisciplinary co-created framework to realise inclusive health AI
Source: PLOS Digit Health. 2026 Sep 23;5(9):e0001096. doi: 10.1371/journal.pdig.0001096 (PMC13600515; doi:10.1371/journal.pdig.0001096)
Supplement: Supplementary file 7 [file pdig.0001096.s008.docx]

**Response to Reviewers**

**Manuscript:** PREFER-IT: A transdisciplinary co-created framework to realise inclusive health AI

Dear Editor,

We thank the editor and the two reviewers for their careful and constructive evaluation of our manuscript. The comments have substantially improved the clarity, framing, and practical value of the work. We were especially glad to see that both reviewers judged the manuscript to be technically sound, ethically rigorous, and clearly written, and that they considered the data to support our conclusions. Below, we respond to each point in turn. For clarity, the original comments are reproduced in italics, and our responses follow in plain text. All corresponding changes have been incorporated into the revised manuscript and are visible in the tracked-changes version (page and line number correspond to the tracked-changes version).

**To the Editor**

We are grateful for your assessment that PREFER-IT addresses a genuine gap and that the co-creation methodology, the mapping of thematic clusters onto structural layers and lifecycle phases constitute a meaningful contribution. We address each of your points below.

Terminology: “medical” vs “health”

*The most important concern is terminology. Using “medical AI” and “medical inclusivity” throughout risks excluding community-based, public health, and non-clinical contexts. Please either adopt “health AI” and “health inclusivity” as your primary terms or provide a substantive rationale for retaining “medical,” and acknowledge this choice as a limitation.*

**Response:** We have adopted the suggestion of the reviewer to use the terms “health AI” and “health inclusivity” as the primary terms throughout the manuscript, instead of the term “medical”. To preserve transparency about the workshop’s original vocabulary and the rationale for this change, we added an explanatory footnote: “During the workshop, we used the terms “medical AI” and “medical inclusivity”. Specifying “medical” rather than “health” did not limit the breadth of the discussion or the topics addressed, though realising the limitations of the word “medical” and following reviewer feedback, in alignment with PLOS Digital Health's broader framing, we revised these to “health AI” and “health inclusivity” throughout the manuscript, to include public health, population health, community health, non-clinical contexts, and other settings where underserved populations most frequently encounter AI systems.” *(page 12, after line 309).* The phrase “Inclusive Medical AI” is retained only where it refers to the workshop (“Inclusive Medical AI: Exploring Diverse Perspectives”), since this was the formal title of the workshop and cannot be altered.

Geographic scope

*Please address geographic scope more explicitly in the abstract and introduction. The workshop was conducted in the Netherlands with a predominantly European participant pool, and many anchors are Europe-specific. Global applicability should be reframed as a future validation goal rather than a present claim.*

**Response:** We have made the European and Dutch context more explicit at every relevant point. The abstract now states that the framework “is based on the outcomes of a five-day transdisciplinary co-creation workshop … held in the Netherlands.” *(page 5, line 95).* The author summary now states that “During a five-day co-creation workshop in the Netherlands…” *(page 6, line 122)*. The final paragraph of the introduction now reads: “Developed through a transdisciplinary co-creation workshop held in the Netherlands, involving mostly European researchers and reflecting multiple disciplines and stakeholder perspectives, including patients, PREFER-IT provides a practical framework to realise health AI inclusivity.” *(page 10, line 240).* The Methods section also clarifies that the participants have a European background “The event brought together 37 experts, from different European countries, …” *(page 10, line 257).*

Consistent with this framing, the discussion presents global relevance as a future validation goal rather than a present claim, describing the European setting as “a strong basis for extending and validating the framework in other settings” and identifying cross-context testing and adaptation as a next step to ensure “global relevance and applicability.” *(page 34, lines 944-947).*

AI-generated imagery in S4 Figure

*Reviewer 2 raises a concern that S4 Figure may contain AI-generated imagery. Please verify and, if confirmed, add disclosure to the AI use declaration section.*

**Response:** We confirm that the icons incorporated into the graphical abstract (S4 Figure) were generated with Microsoft Copilot and subsequently selected and integrated into a custom design by the authors. We have added the following sentence to the Declaration of Generative AI and AI-assisted technologies section: “Microsoft Copilot was used to generate icons incorporated into S4 Figure. These AI-generated visual elements were selected and integrated into a custom graphical abstract design.” *(page 18, lines 444-446).*

Presentation of Table 2

*Table 2’s current layout buries the nuanced problem statements it contains; consider whether some content could be narrated in surrounding text with the table serving a more selective summary function.*

**Response:** We have restructured Table 2 (*page 22*) so that it now presents a condensed summary of the problem clusters, core problem framing, and principal stakeholders for each challenge. The full point-of-view problem statements that were previously embedded in the table are now narrated in the surrounding text of the Definition section *(pages 19-20, lines 466-496),* and consolidated, together with the complete stakeholder maps, in the new S2 Table *(page 50).* The table legend marks this relocation so readers can locate the complete statements without the summary being overloaded.

Colour scheme of Figure 3

*The red-to-orange colour gradient may inadvertently suggest a priority ordering among PREFER-IT elements. Please revise the colour scheme or add a clarifying note.*

**Response:** We have replaced the previous colour scheme of Fig 3 *(page 24)* with the eight-colour Okabe–Ito/Wong qualitative palette (Okabe & Ito, 2002; Wong, 2011), a categorical palette specifically designed to avoid any implied ordering and to remain unambiguous for both colour-blind and non-colour-blind readers. The change has been carried through consistently to Fig 4 *(page 31)*, Fig 5 *(page 32)*, and S4 Figure *(page 53)*. We have also added the following note to Figure 3: “The colours of PREFER-IT follow the 8-colour Okabe-Ito palette and are used for categorical identification only, with no implied ordering or priority.” *(page 24, line 743).*

Methodological transparency on patient contributions

*Please provide greater transparency around patient contributions. It is not clear how patient expert input during Day 2 visibly influenced the ideation and prototype stages. Two or three concrete examples of how patient perspectives shifted expert consensus would substantiate the participatory claims and respond to the tokenism concern.*

**Response:** We have made two complementary changes. First, in the Methods, we now clarify that the Day 2 patient co-creation outputs (personas and journey maps) were not treated as a discrete exercise: they were kept as a constant reference on each group’s work board and explicitly reviewed during Day 3 (ideation) and Day 4 (prototyping), with facilitators prompting groups to use patient-derived needs as evaluation criteria *(page 17, lines 424-428*). Second, in the Results, we added three concrete examples tracing how patient input modified the group problem statements and shaped specific prototype solutions *(pages 26-27, lines 795-808)*. We have explicitly linked these examples to the tokenism discussion, framing the traceable influence of patient input as evidence that participation moved beyond symbolic presence, while noting that a single workshop cannot substitute for sustained, reflexive engagement *(page 34, lines 960-963)*.

Practical guidance for using PREFER-IT

*The discussion identifies important systemic gaps but offers limited guidance on how PREFER-IT would be used to navigate them in practice. Some forward-looking discussion of what adopting this framework would concretely look like for a research team or institution would strengthen the practical contribution.*

**Response:** We have added a passage to the Discussion describing concrete adoption at two levels *(pages 35-37).*

For a team, the dual-axis map (Fig 4) is presented as a planning canvas to be used at project inception: for each intersection of structural layer and AI lifecycle phase, the team asks which PREFER-IT requirements apply, what actions they imply, and who is accountable, assigning ownership of each element to a named role and revisiting the map at each AI lifecycle transition *(pages 35-36, lines 994-1006).*

At the institutional level, we describe how PREFER-IT could be embedded within existing governance checkpoints (research ethics review, data-protection impact assessments, clinical validation gates, and procurement and funding criteria), so that inclusivity is examined alongside safety and efficacy. *(page 37, lines 1026-1035)*

We have explicitly linked these practices to the systemic gaps identified earlier in the Discussion, including the standardisation-value-pluralism tension, the invocation of ethical principles without their true operationalisation and the risk of tokenism *(pages 36-37, lines 1007-1025).*

**Response to Reviewer #1**

We thank Reviewer #1 for the thorough and encouraging assessment, and in particular for affirming that the manuscript is technically sound, that the analysis was performed appropriately, and that the data support the conclusions. We respond to the substantive suggestions below.

Greater detail on the day-to-day workshop process and decision-making

*One point of critique … is more clearly outlining the in-person workshop’s findings from day to day with greater detail on what occurred … what is lacking is the nuance behind this decision-making … ensuring a comprehensive exposition on how these were navigated in the daily processes of the workshop itself would help in improving the soundness of findings.*

**Response:** We have strengthened the description of how the workshop unfolded across days and how patient-centred considerations were carried through the decision-making process. The Methods now make explicit that the Day 2 patient personas and journey maps remained a constant reference on each group’s board and were revisited during ideation (Day 3) and prototyping (Day 4), with patient-derived needs functioning as evaluation criteria *(page 17, lines 424-428*). The Results now trace concrete instances in which patient input reshaped the group problem statements and the resulting solutions *(pages 26-27, lines 795-808)*. Together with the existing discussion of authentic participation and the risk of tokenism *(page 34, lines 960-963)*, these additions make the daily navigation of these tensions more transparent and, we hope, address the reviewer’s concern about the nuance behind the decision-making *(also answered to Editor on Methodological transparency on patient contributions*).

Readability of Table 2

*A clearer visual for Table 2 outlining findings in a more readable manner … the current display does not allow these points to be highlighted due to the influx of information presented … a distinction between every point, such as further breaking down each section or consolidating verbiage, will allow for a firmer grasp.*

**Response:** We agree, and this concern aligns with the editor’s comment on Table 2. We have condensed Table 2 (*page 22*) to present only the problem clusters, core problem framing, and principal stakeholders for each challenge, and have moved the full point-of-view problem statements into the narrative of the Definition section *(pages 19-20, lines 466-496*, with the complete statements and stakeholder maps consolidated in the new S2 Table *(page 50)*. This makes the central findings easier to read while preserving full detail for interested readers. *(also answered the Editor on Presentation of Table 2).*

Emphasis on the “humanity” of health AI tools

*More emphasis on the “humanity” of such tools could help … the question of what would lead a patient to prefer medical AI as opposed to the traditional healthcare professional … is there a certain level of trust needed in AI as a whole to reach this and minimise such power dynamics?*

**Response:** We appreciate this reflection. The framework foregrounds the human and relational dimensions of inclusive health AI through the Participatory and co-design (P) and Inclusive communication (I) elements, and through the centrality of the “humans” pillar in the dual-axis model, which emphasises stakeholder involvement and diverse representation across the lifecycle. The Discussion addresses trust and power asymmetries directly in its treatment of authentic participation, proxies, and the risk of tokenism, and the added practical-adoption passage stresses involving patient representatives in defining research questions and evaluation metrics rather than consulting them after key decisions are made. We have kept the framing centred on inclusivity and meaningful participation, which we believe is the most defensible way to address the trust and relational concerns the reviewer raises, while avoiding empirical claims about patient preferences between AI and human professionals that fall outside the scope of this co-creation study.

**Response to Reviewer #2**

We thank Reviewer #2 for the focused and helpful comments, which we have addressed in full. Several overlap with the editor’s points; we summarise the changes here and cross-reference where relevant.

Terminology: “health inclusivity” and “inclusive health AI”

*The terms “medical inclusivity” and “inclusive medical AI” are not inclusive … The term you should use is “health inclusivity” and “inclusive health AI.” … Using “medical” also excludes those in the communities where underserved populations exist.*

**Response:** We have adopted “health inclusivity” and “inclusive health AI” as the primary terms and added a footnote *(page 12, after line 309)* explaining the change and its rationale. “Inclusive Medical AI” is retained only as the formal name of the workshop *(also answered the Editor on Terminology: “medical” vs “health”).*

Global vs the Netherlands scope

*Are you developing this as a global framework or within the Netherlands? This should be discussed in the introduction with justifications.*

**Response:** We have clarified that the framework was developed in the Netherlands within a predominantly European context. This is now stated explicitly in the abstract *(page 5, line 95)*, the author summary *(page 6, line 122)*, and the final paragraph of the introduction *(page 10, line 240).* The discussion frames cross-context testing and adaptation as the next step toward global relevance *(page 34, lines 944-947) (also answered the Editor on* *Geographic scope).*

Colour of Figure 3

*The colour used for Figure 3 might unintentionally indicate severity (red as worst). It is also difficult to read due to poor contrast.*

**Response:** We have adopted the eight-colour Okabe–Ito/Wong categorical palette. The new palette is applied consistently across Fig 3 (*page 24)*, Fig 4 *(page 31)*, Fig 5 *(page 32)*, and S4 Figure *(page 53)*, and a clarifying note has been added to Fig 3 *(page 24, line 743)* *(also answered the Editor on* *Colour scheme of Figure 3).*

AI-generated images in S4 Figure

*S4 Figure seems to have AI-generated images. There needs to be part of the declaration.*

**Response:** Confirmed and addressed *(page 18, lines 444-446)*. *(also answered the Editor on* AI-generated imagery in S4 Figure*).*

**Journal requirements and copyright queries**

Competing interests

**Response:** The authors have declared that no competing interests exist. This is also stated in the manuscript under “Competing interests.” *(page 54, lines 1298-1302)*.

Ethics statement

**Response:** The Ethics statement now appears in its entirety at the beginning of the Methods section under a dedicated “Ethics statement” subheading. *(page 10, lines 245-252)*.

Financial disclosure

**Response:** The Financial disclosure statement has been updated accordingly *(page 54, lines 1286-1297).*

Data availability

**Response:** We have revised the Data availability statement to explain the basis for the sharing restriction *(pages 54-55, lines 1303-1328).* The data (anonymised workshop notes, co-created personas, and patient journey maps) cannot be shared publicly because the small number of participants and the rich qualitative detail create a residual risk of re-identification despite anonymisation. The restriction was not imposed by a Research Ethics Committee or Institutional Review Board, but reflects the informed consent provided by participants (who consented to the dissemination of anonymised, aggregated insights only) and the data-protection requirements of UMCG. Access requests may be submitted to UMCG’s Data Protection Officers (privacy@umcg.nl).

Copyright – S2 Figure

**Response:** S2 Figure is adapted from Wang and Blok’s open-access publication, distributed under the Creative Commons Attribution 4.0 International License. This is already documented in the manuscript’s Licences and copyright section. *(page 55, lines 1329-1337).*

Copyright – S4 Figure

**Response:** The icons in S4 Figure were generated using Microsoft Copilot, as now disclosed in the Declaration of Generative AI and AI-assisted technologies section. They were not hand-drawn by the authors nor sourced from a third-party clip-art library; they were produced by the generative AI tool and then selected and integrated into a custom design by the authors. We confirm that none of the generated icons replicates identifiable copyrighted characters, trademarked logos, or existing branded artwork. Should the editorial office prefer a more conservative approach, given the evolving copyright status of AI-generated content, we are willing to replace these icons with equivalents from Wikimedia Commons or Openclipart.

We believe the revisions have meaningfully strengthened the manuscript, and we are grateful to the editor and reviewers for their guidance. We hope the revised version is now suitable for publication in PLOS Digital Health and look forward to your decision.

Yours sincerely,

**Patrícia Pita Ferreira, on behalf of all co-authors**

Corresponding author (p.pita.ferreira@umcg.nl)
