# Supplementary material for: PREFER-IT: A transdisciplinary co-created framework to realise inclusive health AI
Source: PLOS Digit Health. 2026 Sep 23;5(9):e0001096. doi: 10.1371/journal.pdig.0001096 (PMC13600515; doi:10.1371/journal.pdig.0001096)
Supplement: Peer review history

# Peer review: Decision Letter 0

**DOI:** 10.1371/journal.pdig.0001096.r001

**Authors:** Po-Chih Kuo, Milit Patel

8 Jun 2026

Response to Reviewers Revised Manuscript with Track Changes Manuscript **Journal Requirements:**

1. Please send a completed 'Competing Interests' statement, including any COIs declared by your co-authors. If you have no competing interests to declare, please state "The authors have declared that no competing interests exist". Otherwise please declare all competing interests beginning with the statement "I have read the journal's policy and the authors of this manuscript have the following competing interests:"

2. Please ensure that your Ethics Statement is available in its entirety at the beginning of your Methods section, under a subheading 'Ethics Statement'.

3. Please amend your detailed Financial Disclosure statement. This is published with the article. It must therefore be completed in full sentences and contain the exact wording you wish to be published.

a. State the initials, alongside each funding source, of each author to receive each grant.

4. We note that you have indicated that there are restrictions to data sharing for this study. For studies involving human research participant data or other sensitive data, we encourage authors to share de-identified or anonymized data. However, when data cannot be publicly shared for ethical reasons, we allow authors to make their data sets available upon request. For information on unacceptable data access restrictions, please see http://journals.plos.org/plosone/s/data-availability#loc-unacceptable-data-access-restrictions.

Before we proceed with your manuscript, please address the following prompts:

a) If there are ethical or legal restrictions on sharing a de-identified data set, please explain them in detail (e.g., data contain potentially identifying or sensitive patient information, data are owned by a third-party organization, etc.) and who has imposed them (e.g., a Research Ethics Committee or Institutional Review Board, etc.). Please also provide contact information for a data access committee, ethics committee, or other institutional body to which data requests may be sent.

b) If there are no restrictions, please upload the minimal anonymized data set necessary to replicate your study findings to a stable, public repository and provide us with the relevant URLs, DOIs, or accession numbers. Please see http://www.bmj.com/content/340/bmj.c181.long for guidelines on how to de-identify and prepare clinical data for publication. For a list of recommended repositories, please see https://journals.plos.org/plosone/s/recommended-repositories. You also have the option of uploading the data as Supporting Information files, but we would recommend depositing data directly to a data repository if possible.

Please update your Data Availability statement in the submission form accordingly.

5. Some material included in your submission may be copyrighted. According to PLOS’s copyright policy, authors who use figures or other material (e.g., graphics, clipart, maps) from another author or copyright holder must demonstrate or obtain permission to publish this material under the Creative Commons Attribution 4.0 International (CC BY 4.0) License used by PLOS journals. Please closely review the details of PLOS’s copyright requirements here: PLOS Licenses and Copyright. If you need to request permissions from a copyright holder, you may use PLOS's Copyright Content Permission form.

Please respond directly to this email or email the journal office and provide any known details concerning your material's license terms and permissions required for reuse, even if you have not yet obtained copyright permissions or are unsure of your material's copyright compatibility.

Potential Copyright Issues:

"S2Fig.tif" and "S4Fig.tif": Please confirm whether you drew the images / clip-art within the figure panels by hand. If you did not draw the images, please provide (a) a link to the source of the images or icons and their license / terms of use; or (b) written permission from the copyright holder to publish the images or icons under our CC-BY 4.0 license. Alternatively, you may replace the images with open source alternatives. See these open source resources you may use to replace images / clip-art:

- https://commons.wikimedia.org

- https://openclipart.org/

**Additional Editor Comments (if provided):**

The most important concern is terminology. Using "medical AI" and "medical inclusivity" throughout risks excluding community-based, public health, and non-clinical contexts where underserved populations most frequently encounter AI systems. This is in tension with PLOS Digital Health's own deliberate move beyond the "medical" framing. Please either adopt "health AI" and "health inclusivity" as your primary terms or provide a substantive rationale in the introduction for retaining "medical," and acknowledge this choice as a limitation.

Please also address geographic scope more explicitly in the abstract and introduction. The workshop was conducted in the Netherlands with a predominantly European participant pool, and many solutions and regulatory anchors are Europe-specific. Global applicability should be reframed as a future validation goal rather than a present claim.

Reviewer 2 raises a concern that S4 Figure may contain AI-generated imagery. Please verify and, if confirmed, add disclosure to the AI use declaration section.

On presentation, please revisit Table 2 and Figure 3. Table 2's current layout buries the nuanced problem statements it contains; consider whether some content could be narrated in surrounding text with the table serving a more selective summary function. For Figure 3, the red-to-orange color gradient may inadvertently suggest a priority ordering among PREFER-IT elements. Please revise the color scheme or add a clarifying note.

We also ask for greater methodological transparency around patient contributions specifically. While participant diversity is well-described, it is not clear how patient expert input during Day 2 visibly influenced the ideation and prototype stages. Two or three concrete examples of how patient perspectives shifted expert consensus would substantiate the paper's participatory claims and respond directly to the tokenism concern you raise in the discussion.

**Reviewers' Comments:**

**Comments to the Author**

1. Does this manuscript meet PLOS Digital Health’s publication criteria?

Reviewer #1: Yes

Reviewer #2: Yes

**********

2. Has the statistical analysis been performed appropriately and rigorously?

Reviewer #1: Yes

Reviewer #2: N/A

**********

3. Have the authors made all data underlying the findings in their manuscript fully available (please refer to the Data Availability Statement at the start of the manuscript PDF file)?

The PLOS Data policy

Reviewer #1: Yes

Reviewer #2: Yes

**********

4. Is the manuscript presented in an intelligible fashion and written in standard English?

Reviewer #1: Yes

Reviewer #2: Yes

**********

Reviewer #1: “PREFER-IT: A transdisciplinary co-created framework to realise inclusive medical AI” is a research article curated to address the rising questions regarding the implementation of medical AI across various sectors of society differing in socioeconomic status, culture, etc. This provides a framework in which this expansion of medical AI can remain ethical and not further swell existing inequalities that may appear in healthcare today. Following an immersive workshop with experts in healthcare, ethics, law, social sciences, AI, and patient advocacy, PREFER-IT was created along with key specifics on what the initial drawbacks of AI may be without the proper care taken to ensure its implementation is delivered efficiently and fairly across all populations.

This article, following data collection and interpretation of results, remains valid with statistical bounds clearly stated and those within it performed. As far as the connection between the two, there is a clear relationship that the reader is able to understand at the time their own questions may be raised as these findings are new to the world of artificial intelligence and how this, in healthcare, is able to abide by both the guidelines/protocol current experts do as well as ethics that humans inherently carry out. This does, however, lead to the question of what would lead a patient to prefer medical AI as opposed to the traditional healthcare professional: is there a certain level of personability that can only be found in humans? Even if, on record, this bridge could be gapped, is there a certain level of trust needed in AI as a whole to reach this and minimize such power dynamics? The introduction does exceptionally well addressing these concerns, although more emphasis on the “humanity” of such tools could help assist in the overarching theme that, with PREFER-IT’s mission and meaning, the benefits of the expansion in medical AI could provide advantages that not only trump that of current logistic healthcare limitations but match the mutual communication a patient is able to build with that of whoever is treating them.

One point of critique within the zone of general data collection is more clearly outlining the In-Person workshop's findings from day to day and with greater detail on what occurred (such as remarks of personal accounts from participants, features of the environment leading to decision-making, etc.) as these appear slightly vague but retain great value in subsequent areas. Importantly noted are the credentials of participants, the unbiased collection of data, etc., but what is lacking is the nuance behind this decision-making. Does the curse of knowledge impact the practicality of conclusions found in definition, ideation, prototype, and prioritise that contributors were able to brainstorm? Does this reflect the true best will of the patients living in such communities aimed to be served, rather than operational efficiency? Lines 595-601 address similar concerns, but ensuring a comprehensive exposition on how these were navigated in the daily processes of the workshop itself would help in improving the soundness of findings. Additionally, while Table 4 provides a summary of shared priorities and gaps that address such notions, explaining how these concepts had implications on the processes of the workshop and how each day brought about new, or potentially the same, general themes in greater detail would allow for more of an understanding as to the relatability the consensus these experts had is to the public.

A clearer visual for Table 2 outlining findings in a more readable manner, will in addition allow for greater reader digestion of how these ideas all coincide to contribute to the overall goal of PREFER-IT. In particular, this table conveys crucial specifics in the discoveries of the participant majority regarding three pivotal scenarios in the implementation of AI, how to acknowledge and consolidate these problems, and potential actions that may be taken in regards to addressing these on a larger, realistic scale. But, the current display of this table does not allow these points to be highlighted due to the influx of information presented in the space as opposed to taking an approach in which this information is embedded in its surrounding text and subsequently rooted from this. A distinction between every point, such as further breaking down each section or consolidating verbiage, will allow for a firm grasp of the specific ways in which concerns that have arisen regarding medical AI could be remedied and the recognition that they are acknowledged in the first place.

As a reader, it is very crucial to conceptualize the implications of AI on the medical field and how these specific points of concern require deep consideration, something this research article does outstandingly well on. Utilizing this message that the inclusivity of medical AI starts with those involved in the framework, not simply the outcome, allows for a certain level of trust one is able to put in new technologies seeping into significant sectors of society such as healthcare. Immersing this theme into every section, as has been done, will allow for a higher degree of connection between concept and effect in this new world of linking innovation to society.

Reviewer #2: See comments/suggestions

1. The irony for this paper is that the terms "medical inclusivity" and "inclusive medical AI" are not inclusive in the sense that your definition talks about inclusivity in all aspects of "health." This journal also uses the term "Digital Health" because of the limitations with using the term "medical." The term you should use is "health inclusivity" and "inclusive health AI" This goes along the same historical issue about electronic medical records and electronic health records (EHR is a more inclusive term). Using the term "medical" also excludes those in the communities where underserved populations exists. These are the people whom we need to include since they will be the ones to experience inequality since AI use contributes to pre-existing inequalities in healthcare.

2. Are you developing this as a global framework or within the Netherlands? This should be discussed in the introduction with justifications.

3. The color used for Figure 3 might unintentionally indicate severity (red as worst). It is also difficult to read due to poor contrast.

4. S4 Figure seems to have AI generated images. There needs to be part of the declaration.

**********

what does this mean?). If published, this will include your full peer review and any attached files.

**Do you want your identity to be public for this peer review?**  If you choose “no”, your identity will remain anonymous but your review may still be made public.

For information about this choice, including consent withdrawal, please see our Privacy Policy

Reviewer #1: **Yes:** Alexandra Rivera

Reviewer #2: No

**********

**Figure resubmission:**

**Reproducibility:**

To enhance the reproducibility of your results, we recommend that authors of applicable studies deposit laboratory protocols in protocols.io, where a protocol can be assigned its own identifier (DOI) such that it can be cited independently in the future. Additionally, PLOS ONE offers an option to publish peer-reviewed clinical study protocols. Read more information on sharing protocols at https://plos.org/protocols?utm_medium=editorial-email&utm_source=authorletters&utm_campaign=protocols

# Author comment: Author response to Decision Letter 1

**DOI:** 10.1371/journal.pdig.0001096.r002

22 Jul 2026

Attachment Submitted filename: 01072026_letter_reviewers.docx

# Peer review: Decision Letter 1

**DOI:** 10.1371/journal.pdig.0001096.r003

**Authors:** Po-Chih Kuo, Milit Patel

27 Aug 2026

PREFER-IT: A transdisciplinary co-created framework to realise inclusive health AI

PDIG-D-25-00955R1

Dear Dr. Pita Ferreira,

We are pleased to inform you that your manuscript 'PREFER-IT: A transdisciplinary co-created framework to realise inclusive health AI' has been provisionally accepted for publication in PLOS Digital Health.

Before your manuscript can be formally accepted you will need to complete some formatting changes, which you will receive in a follow-up email from a member of our team.

Please note that your manuscript will not be scheduled for publication until you have made the required changes, so a swift response is appreciated.

IMPORTANT: The editorial review process is now complete. PLOS will only permit corrections to spelling, formatting or significant scientific errors from this point onwards. Requests for major changes, or any which affect the scientific understanding of your work, will cause delays to the publication date of your manuscript.

If your institution or institutions have a press office, please notify them about your upcoming paper to help maximize its impact. If they'll be preparing press materials, please inform our press team as soon as possible -- no later than 48 hours after receiving the formal acceptance. Your manuscript will remain under strict press embargo until 2 pm Eastern Time on the date of publication. For more information, please contact digitalhealth@plos.org.

Thank you again for supporting Open Access publishing; we are looking forward to publishing your work in PLOS Digital Health.

Best regards,

Milit Patel

Guest Editor

PLOS Digital Health

***********************************************************

**Additional Editor Comments (if provided):**

Thank you for your thorough revisions. Both reviewers confirm that all previously raised comments have been fully addressed and recommend acceptance without further changes. The manuscript was judged to meet all PLOS Digital Health publication criteria, including methodological rigor, appropriate statistical analysis, full data availability, and clear writing. No further revisions are required. Congratulations on this outcome.

**Reviewer Comments (if any, and for reference):**

Reviewer's Responses to Questions

**Comments to the Author**

Reviewer #2: All comments have been addressed

Reviewer #3: All comments have been addressed

**********

publication criteria?

Reviewer #2: Yes

Reviewer #3: Yes

**********

3. Has the statistical analysis been performed appropriately and rigorously?

Reviewer #2: Yes

Reviewer #3: Yes

**********

4. Have the authors made all data underlying the findings in their manuscript fully available (please refer to the Data Availability Statement at the start of the manuscript PDF file)?

The PLOS Data policy

Reviewer #2: Yes

Reviewer #3: Yes

**********

5. Is the manuscript presented in an intelligible fashion and written in standard English?

Reviewer #2: Yes

Reviewer #3: Yes

**********

Reviewer #2: Thank you for addressing all comments/feedback. Congratulations!

Reviewer #3: (No Response)

**********

what does this mean?). If published, this will include your full peer review and any attached files.

**Do you want your identity to be public for this peer review?**  If you choose “no”, your identity will remain anonymous but your review may still be made public.

For information about this choice, including consent withdrawal, please see our Privacy Policy

Reviewer #2: **Yes:** John Robert Bautista

Reviewer #3: **Yes:** Vitalii Poberezhets

**********
